# Molecularly engineered AIEgens with enhanced quantum and singlet-oxygen yield for mitochondria-targeted imaging and photodynamic therapy†

**DOI:** 10.1039/d2sc00889k

**Published:** 2022-08-03

**Authors:** Fang-Zhou Xu, Ling Zhu, Hai-Hao Han, Jian-Wei Zou, Yi Zang, Jia Li, Tony D. James, Xiao-Peng He, Cheng-Yun Wang

**Affiliations:** Key Laboratory for Advanced Materials, Joint International Research Laboratory of Precision Chemistry and Molecular Engineering, Feringa Nobel Prize Scientist Joint Research Center, Frontiers Center for Materiobiology and Dynamic Chemistry, School of Chemistry and Molecular Engineering, East China University of Science and Technology 130 Meilong Rd. Shanghai 200237 China xphe@ecust.edu.cn cywang@ecust.edu.cn; NingboTech University Ningbo 315100 Zhejiang PR China jwzou@nit.net.cn; National Center for Drug Screening, State Key Laboratory of Drug Research, Shanghai Institute of Materia Medica, Chinese Academy of Sciences Shanghai 201203 China jli@simm.ac.cn; University of Chinese Academy of Sciences No. 19A Yuquan Road Beijing 100049 P. R. China; Department of Chemistry, University of Bath Bath BA2 7AY UK t.d.james@bath.ac.uk; School of Chemistry and Chemical Engineering, Henan Normal University Xinxiang 453007 China; Shandong Laboratory of Yantai Drug Discovery, Bohai Rim Advanced Research Institute for Drug Discovery Shandong 264117 Yantai P. R. China

## Abstract

Luminogens characteristic of aggregation-induced emission (AIEgens) have been extensively exploited for the development of imaging-guided photodynamic therapeutic (PDT) agents. However, intramolecular rotation of donor–acceptor (D–A) type AIEgens favors non-radiative decay of photonic energy which results in unsatisfactory fluorescence quantum and singlet oxygen yields. To address this issue, we developed several molecularly engineered AIEgens with partially “locked” molecular structures enhancing both fluorescence emission and the production of triplet excitons. A triphenylphosphine group was introduced to form a D–A conjugate, improving water solubility and the capacity for mitochondrial localization of the resulting probes. Experimental and theoretical analyses suggest that the much higher quantum and singlet oxygen yield of a structurally “significantly-locked” probe (LOCK-2) than its “partially locked” (LOCK-1) and “unlocked” equivalent (LOCK-0) is a result of suppressed AIE and twisted intramolecular charge transfer. LOCK-2 was also used for the mitochondrial-targeting, fluorescence image-guided PDT of liver cancer cells.

## Abstract 2

Luminogens characteristic of aggregation-induced emission (AIEgens) have been engineered for the development of imaging-guided photodynamic therapeutic (PDT) agents.

## Graphical abstract

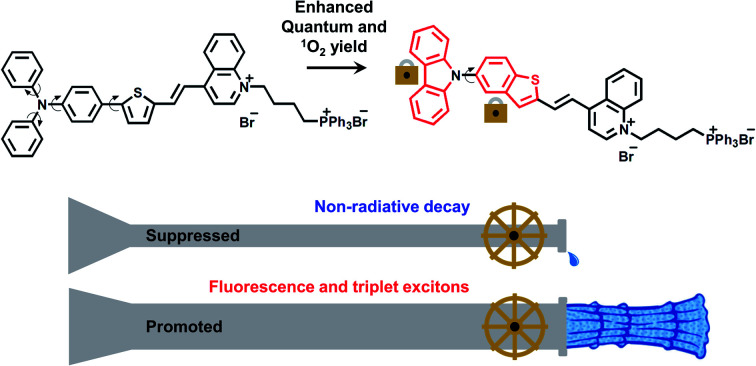

## Introduction

Due to its sensitivity, biocompatibility, and high spatiotemporal precision, fluorescence imaging (FI) is a popular technique for the real-time tracking of the dynamic biological events within cells.^1–5^ In recent years, FI-guided photodynamic therapeutic (PDT) agents and materials have been developed for cancer therapy and exhibit significantly reduced side effects that are typically associated with traditional chemo- and radiotherapeutic drugs.^6–11^ This is because the incorporation of fluorescence and PDT into one single molecule can enable the positional monitoring in cells and *in vivo*, where cytotoxicity is only activated when an external, non-invasive light source is applied. Among all types of FI and PDT-active agents, organic dyes are advantageous in terms of the readily tunable photophysical properties using simple structural modifications.^12–18^ However, most of the reported organic dyes suffer from aggregation-caused quenching (ACQ) in both fluorescence and singlet-oxygen yield, and non-selective localization in cells. Aggregation-induced emission (AIE) was first reported by the Tang group,^19^ and describes the enhanced fluorescence of organic dyes in their aggregated states. Significantly, such AIE-active dyes have exhibited great promise in the field of biomedicine.^20–23^ Luminogens characteristic of AIE (AIEgens) emit strong fluorescence in the aggregated state with high photostability, which is ideal for cell-based and *in vivo* optical imaging.^24–29^ In addition, appropriately designed AIEgens, exhibit superior photosensitization of molecular oxygen than common ACQ-based photosensitizers,^8,30–32^ thereby offering scope for the development of enhanced FI-guided PDT agents.

**Scheme 1:**
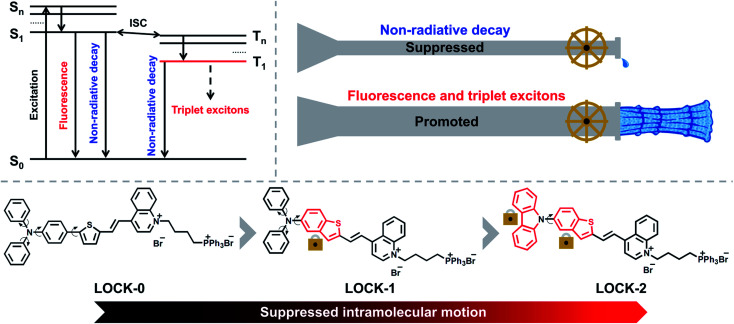
Schematic illustration of the different dissipation pathways of excited AIEgens, and the molecular-engineering strategy of LOCK-0, LOCK-1 and LOCK-2 in order to reduce intramolecular motions, leading to suppressed non-radiative decay from their excited states.

The quantum yield (QY) and reactive-oxygen species (ROS) yield are two main requirements for AIEgens used as FI-guided PDT agents. As depicted in Scheme 1, when AIEgens are excited from the ground state (S_0_) to the singlet state (S_1_ to S_*n*_), there are three pathways for the excited-state photonic energy to dissipate, which include non-radiative decay, fluorescence emission, and intersystem crossing (ISC). The excited-state energy is converted to heat through the non-radiative channel, while, triplet excitons can be released in the form of phosphorescence or can be transferred to other substrates, such as ^3^O_2_, radicals and biomolecules such as DNA to enable PDT.^16,33,34^ The amount of triplet excitons produced determines the theoretical upper limit of the ROS yield. Recently, the Ding group reported an elegant molecular design strategy to improve the photothermal therapy (PTT) efficiency of AIEgens.^35,36^ Long alkyl chains were introduced to the AIEgens in order to enhance the intramolecular motions, and thus the excited-state photonic energy is preferentially harvested through the non-radiative decay pathway, resulting in an improved PTT effect. We sought to employ a contrary strategy that, “locks” the intramolecular motions of common AIEgens, leading to the excited-state photonic energy being preferentially converted to fluorescence emission and triplet excitons.

In this study, three donor–acceptor (D–A) type AIEgens, *i.e.*, LOCK-0, LOCK-1 and LOCK-2, were designed and synthesized (Scheme 1). Aromatic amines and thiophenes were used as electron donors (D), to which quinoline bromide as the electron–acceptor (A) was conjugated. The resulting D–A system was envisioned to have a long fluorescence emission wavelength and a high ROS yield.^32,37–39^ In addition, triphenylphosphine bromide, a known mitochondrion-targeting group, was introduced to the D–A conjugate to improve water-solubility. With our design, the relative motions between the benzene and thiophene ring of LOCK-0 are “locked” to form the benzothiophene derivative LOCK-1. Subsequently, the motions of the phenyl rings in the diphenylamine group of LOCK-1 were further “locked” to form the carbazole derivative LOCK-2. Using this molecular engineering strategy, we envisioned a substantially enhanced QY and ROS yield due to the locked molecular motions resulting in reduced non-radiative decay of the AIEgens.

## Results and discussion

### Synthesis, photophysical and photochemical properties of the AIEgens

**Fig. 1:**
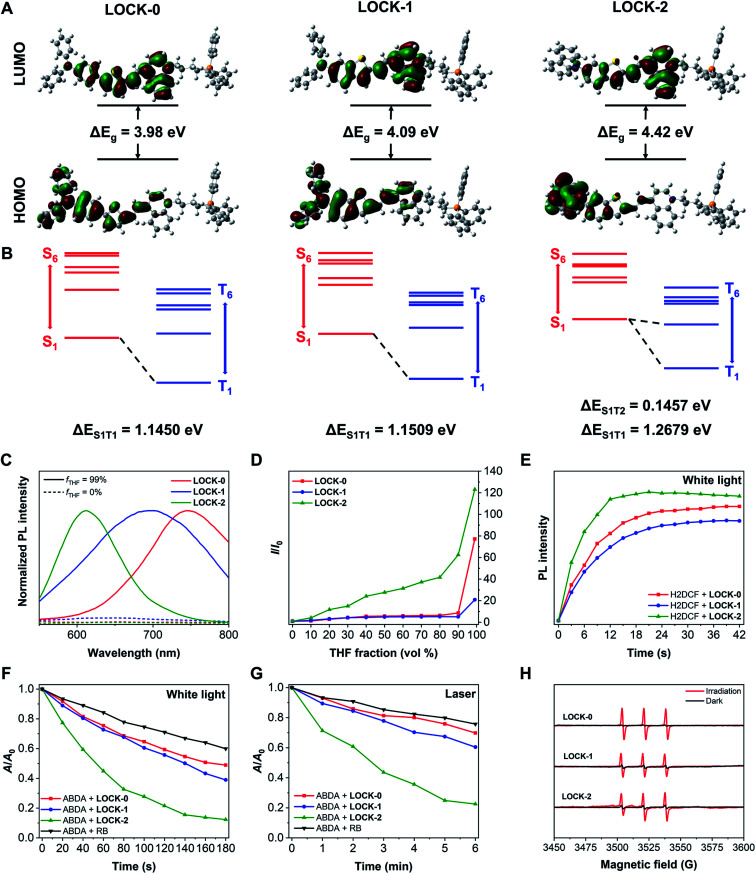
(A) HOMO–LUMO contour plots and Δ*E*_g_ of LOCK-0, LOCK-1 and LOCK-2. (B) Energy level diagram of singlet and triplet states of the three AIEgens. Computational method: CAM-B3LYP/6-31G(d). Solvent: water. (C) Normalized fluorescence intensity of the AIEgens LOCK-0, LOCK1 and LOCK-2 in deionized water (dashed lines) and *f*_T_ = 99% solution (solid lines). AIEgen concentration: 10 μM. *λ*_ex_: LOCK-0 and LOCK-1: 523 nm; LOCK-2: 473 nm. (D) Changes in fluorescence intensity (*I*/*I*_0_, where *I* and *I*_0_ are the fluorescence intensity of an AIEgen in the absence and presence of THF, respectively) of the AIEgens in THF/H_2_O mixtures with different THF fractions. AIEgen concentration: 10 μM. *λ*_ex_: LOCK-0 and LOCK-1: 523 nm; LOCK-2: 473 nm. (E) Fluorescence intensity of H2DCF solution at 523 nm with the AIEgens under white light irradiation with time. AIEgen concentration: 10 μM. H2DCF concentration: 5 μM. White light: 400–700 nm, 10 mW cm^−2^. *λ*_ex_ = 488 nm. (F) Relative absorbance area (*A*/*A*_0_) of ABDA solution at 359, 378 and 399 nm with AIEgens and RB under white light irradiation with time. AIEgen concentration: 5 μM. ABDA concentration: 50 μM. White light: 400–700 nm, 10 mW cm^−2^. (G) Relative absorbance area (*A*/*A*_0_) of ABDA solution at 359, 378 and 399 nm with AIEgens and RB under laser irradiation with time. AIEgen concentration: 5 μM. ABDA concentration: 50 μM. Laser: 520 nm for LOCK-0, LOCK-1 and RB, and 480 nm for LOCK-2, 7 mW cm^−2^. (H) EPR spectra of TEMP with AIEgens in aqueous solution with and without white light irradiation. AIEgen concentration: 1.0 mM. TEMP concentration: 25 mM. White light: 400–700 nm, 10 mW cm^−2^.

LOCK-0, LOCK-1 and LOCK-2 were readily synthesized through the synthetic routes shown in Scheme S1† with a total yield of 50%, 42% and 45%, respectively. Methyl quinoline and triphenyl phosphine (mitochondrion-targeting group) were connected with 1,4-dibromobutane to form an electron–acceptor matrix with two positive charges. A typical electron–donor AIE aldehyde, 5-(4-diphenylamino-phenyl)-thiophene-2-carbaldehyde, was introduced as the “unlocked” moiety of LOCK-0. Diphenylamine and carbazole were connected to benzothiophene using a Buchwald–Hartwig coupling reaction to form the “partially locked” and “locked” electron–donor AIE aldehydes of LOCK-1 and LOCK-2, respectively. LOCK-0, LOCK-1 and LOCK-2 were then assembled using Knoevenagel condensation of the electron–acceptor moiety and corresponding electron–donor AIEgen aldehydes. The UV-visible absorption spectra of the three AIEgens in deionized water are shown in Fig. S1.† The maximum absorption wavelength (*λ*_ex, max_) for the AIEgens is in of the order LOCK-0 = LOCK-1 (523 nm) > LOCK-2 (473 nm) (Table 1). The shorter absorption wavelength of LOCK-2 could be ascribed to the presence of the carbazole group with restricted intramolecular motions and reduced π-conjugation.^40^ Theoretical calculations were performed for the three AIEgens with hybrid density functional, CAM-B3LYP, in combination with 6-31G(d) basis set (Table S1†).^41^ The contour plots of the highest occupied molecular orbital (HOMO) and lowest unoccupied molecular orbital (LUMO) are displayed in Fig. 1A. It was found that the HOMO of LOCK-2 was more delocalized at the carbazole group, and that of LOCK-0 and LOCK-1 was mainly delocalized along the aromatic amines and thiophenes. The HOMO–LUMO energy gaps (Δ*E*_g_) of LOCK-0, LOCK-1 and LOCK-2 were calculated to be 3.98, 4.09 and 4.42 eV, respectively, which is consistent with their maximum absorption wavelengths as determined by UV-vis spectroscopy. The HOMO and LUMO of LOCK-2 is spatially more separated than those of LOCK-0 and LOCK-1, resulting in a disfavored intramolecular charge transfer (ICT) of the former.

**Table 1:** Photophysical and photochemical properties of LOCK-0, LOCK-1 and LOCK-2

AIEgen	*λ*_ex, max_ a (nm)	*λ*_em, max_ b (nm)	Stokes shift^c^ (nm)	*Ε* d (M^−1^ cm^−1^)	QY^e^	^1^O_2_ yield^f^ (white light)	^1^O_2_ yield^g^ (laser)
LOCK-0	523	744	221	16 916	1.23%	111.7%	91.3%
LOCK-1	523	700	177	24 092	1.94%	104.0%	129.7%
LOCK-2	473	612	139	21 667	56.81%	159.1%	205.6%

a Maximum absorption wavelengths of three AIEgens (LOCK-0, LOCK-1 and LOCK-2) in aqueous solution. AIEgen concentration: 10 μM.

b Maximum emission wavelengths of three AIEgens in *f*_T_ = 99% solution. AIEgen concentration: 10 μM. *λ*_ex_: LOCK-0 and LOCK-1: 523 nm; LOCK-2: 473 nm.

c Stokes shifts calculated by *λ*_em, max_^B^ – *λ*_ex, max_^A^.

d Molar extinction coefficients of three AIEgens in aqueous solution. AIEgen concentration: 5 μM.

e QY of three AIEgens in *f*_T_ = 99% solution measured by an Edinburgh FLS980 fluorescence spectrophotometer with a standard integrating sphere. AIEgen concentration: 10 μM. *λ*_ex_: LOCK-0 and LOCK-1: 523 nm; LOCK-2: 473 nm.

f ^1^O_2_ yields calculated with ABDA of three AIEgens under white light irradiation. White light: 400–700 nm, 10 mW cm^−2^. ^1^O_2_ yield of RB = 75%.

g ^1^O_2_ yields calculated with ABDA of three AIEgens under laser irradiation. Laser: 520 nm for LOCK-0, LOCK-1 and RB, and 480 nm for LOCK-2, 7 mW cm^−2^. ^1^O_2_ yield of RB = 75%.

Next, the AIE properties of the AIEgens were evaluated in deionized water with increasing THF fractions (*f*_T_). As shown in Fig. 1C and D and S2,† all the AIEgens displayed minimal fluorescence in water, and their fluorescence was enhanced significantly with 99% *f*_T_, suggesting the good water solubility of the AIEgens. As the *f*_T_ increased from 0% to 99%, the fluorescence intensity of LOCK-2 enhanced gradually, while that of LOCK-0 and LOCK-1 did increase until 99% THF was added. In addition, measuring their solvent sensitivity (Fig. S3†) and Stokes shifts (Table 1) suggests that the twisted intramolecular charge transfer (TICT) effect of the AIEgens is in an order LOCK-0 > LOCK-1 > LOCK-2.^42^ The relatively weaker AIE and TICT effect for LOCK-2 is a result of the suppressed intramolecular motions with respect to LOCK-0 and LOCK-1. The absolute QY of LOCK-0, LOCK-1 and LOCK-2 in *f*_T_ = 99% solution were measured to be 1.23%, 1.94% and 56.81%, respectively (Table 1). We expect that the higher QY of LOCK-2 was due to the suppressed intramolecular motions, facilitating the radiative pathways of the excited-state photonic energy. The dihedral angles (*θ*) of the Csp^2^ (in the terminal benzene)–N–Csp^2^–Csp^2^ were also calculated for the three AIEgens (Fig. S4†). The *θ* values of LOCK-2 at S_0_ and S_1_ states are larger than those of LOCK-0 and LOCK-1, which might reduce intermolecular packing that leads to ACQ. It has been reported that a reduced TICT effect can facilitate an enhanced QY for luminogens,^43,44^ which agrees with our observations with LOCK-2.

2′,7′-Dichloro-dihydrofluorescein (H2DCF), the hydrolyzed product of 2′,7′-dichloro-dihydro-fluorescein diacetate (H2DCF-DA) was used to evaluate the ROS production of the AIEgens. H2DCF is oxidized by ROS to produce 2′,7′-dichloro-fluorescein (DCF) with strong fluorescence detectable at *ca.* 523 nm. As shown in Fig. 1E and S5,† the fluorescence of DCF was observed for all three AIEgens under white light irradiation, proving their capacity for ROS production. The fluorescence intensity of DCF with LOCK-2 increased more significantly than for LOCK-0 and LOCK-1, suggestive of the higher ROS yield for the former. We then quantitatively determined the ROS yields of the AIEgens using 9,10-anthracenediyl-bis(methylene)dimalonic acid (ABDA). The absorption peaks at 359, 378 and 399 nm of ABDA decreases when ^1^O_2_ is present. Two excitation sources, white light (400–700 nm) and a laser (480 or 520 nm), were used. As shown in Fig. 1F and G and S6,† after irradiation with both light sources, the absorption peaks of ABDA decreases rapidly with all the AIEgens, clearly indicating the generation of ^1^O_2_. Using Rose Bengal (RB) as a reference with a 75% yield, the ^1^O_2_ yields of LOCK-0, LOCK- 1 and LOCK-2 were determined to be 111.7%, 104.0% and 159.1% under white light irradiation, and 91.3%, 129.7% and 205.6% under laser irradiation (Table 1 and Fig. S6 and S7†), respectively. The higher ^1^O_2_ yield of LOCK-2 compared with LOCK-0 and LOCK-1 could be partially explained by differences in the energy gap between singlet and triplet excited-states (Δ*E*_ST_). The excited-state characteristics of the three AIEgens were calculated by time-dependent DFT, and the solvent effects were considered by applying the conductor-like polarizable continuum model (CPCM).^41^ The energy gap between the S_1_ and T_2_ excited-states of LOCK-2 was 0.1457 eV (Fig. 1B and Tables S2–S4†), significantly smaller than the energy gaps between the S_1_ and T_1_ excited-states of LOCK-0 (1.1450 eV) and LOCK-1 (1.1509 eV). This means that LOCK-2 probably exhibits an enhanced ISC process, and therefore increased ROS production. Moreover, the higher ^1^O_2_ yield of LOCK-2 could also be ascribed to the reduced intramolecular motions, which is consistent with the enhanced QY when compared with the other two AIEgens. Electron paramagnetic resonance (EPR) with the ^1^O_2_ trapping agent, tetramethylpiperidine (TEMP), was used to corroborate the presence of ^1^O_2_ when the AIEgens were irradiated with light. As shown in Fig. 1H, in all three AIEgens groups, characteristic triple peaks assigned to ^1^O_2_ were recorded after white light irradiation.^8^

### Fluorescence-guided imaging and PDT in HepG2 cells

With their promising optical properties determined, we then evaluated the FI and PDT properties of the AIEgens, LOCK-0, LOCK-1 and LOCK-2 in living cells. Mitochondria are the “energy factory” of cells, and play key roles in cell differentiation, cell communication, cell growth, and apoptosis.^45^ The mitochondrial membrane potential (MMP) of cancer cells has been found to be generally higher than those of normal cells, making mitochondrial-targeting agents suitable for targeted cancer cell localization.^46,47^ Unsatisfactory photostability and the need for washing before imaging are the drawbacks of many existing mitochondrial-targeting fluorescent probes. Due to the excellent water solubility and significant fluorescence enhancement upon aggregation, we evaluated the FI capacity of the AIEgens in a wash-free manner with HepG2 (human hepatoma) as a model cell line.

**Fig. 2:**
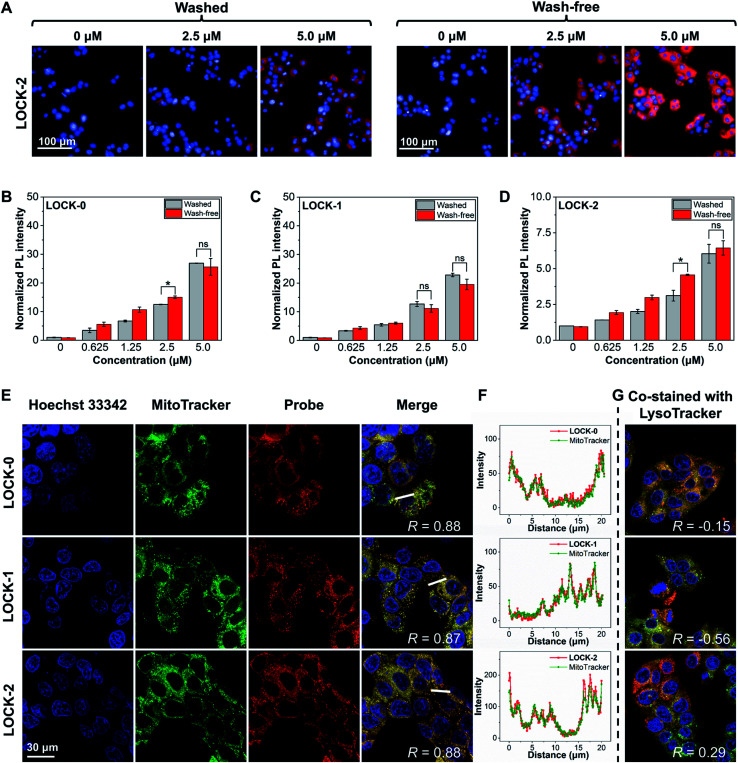
(A) Concentration-dependent fluorescence imaging by confocal laser-scanning microscopy and (B–D) fluorescence quantification of HepG2 cells with LOCK-0, LOCK-1 and LOCK-2 in washed and wash-free manner. Hoechst 33342 concentration: 5 μg mL^−1^. Excitation source: Hoechst 33342: 405 nm; LOCK-0: 562 nm; LOCK-1: 562 nm; LOCK-2: 488 nm. Emission filter: Hoechst 33342: 410–480 nm; LOCK-0: 600–800 nm; LOCK-1: 600–800 nm; LOCK-2: 570–630 nm. (E) Fluorescence imaging of HepG2 cells with LOCK-0, LOCK-1 and LOCK-2 co-stained with MitoTracker® Deep Red (F) fluorescence quantification of a selected section across the HepG2 cells. (G) Fluorescence imaging of HepG2 cells with LOCK-0, LOCK-1 and LOCK-2 co-stained with LysoTracker® Deep Red by confocal laser-scanning microscopy. Hoechst 33342 concentration: 5 μg mL^−1^. MitoTracker® Deep Red, LysoTracker® Deep Red concentration: 200 nM. AIEgens concentration: 5 μM. Excitation source: Hoechst 33342: 405 nm; MitoTracker® Deep Red, LysoTracker® Deep Red: 638 nm; LOCK-0: 562 nm; LOCK-1: 562 nm; LOCK-2: 488 nm. Emission filter: Hoechst 33342: 415–487 nm; MitoTracker® Deep Red, LysoTracker® Deep Red: 648–710 nm; LOCK-0: 600–800 nm; LOCK-1: 600–800 nm; LOCK-2: 570–650 nm. *P < 0.05, ns: not significant.

Concentration-dependent, wash-free experiments of the three AIEgens were conducted to initially assess their FI capability. As illustrated in Fig. S8,† using Hoechst 33342 to stain the cell nucleus, the cells in all the three groups exhibited strong fluorescence corresponding to the AIEgens after incubation for 1 h. The fluorescence intensities of the AIEgens in both wash-free and washed groups were enhanced as the concentration of the probes increased (such as LOCK-2 in Fig. 2A). In addition, the fluorescence intensities of the AIEgens in the two groups were found to be similar, suggesting that minimal background fluorescence is produced with the wash-free protocol (Fig. 2B–D). This observation agrees with the AIE feature of the water-soluble probes, which show minimal fluorescence in highly aqueous solutions (such as the cytoplasm), where their fluorescence is only enhanced when they are aggregated or rigidified. In addition, all three AIEgens exhibited good photostability after continuous laser-scanning (60 times, Fig. S9 and S10†).

To determine whether the fluorescence in cells was the result of the aggregation of the probes in the mitochondria, a co-localization assay was carried out. MitoTracker and LysoTracker, which selectively stain the mitochondria and lysosomes in cells, respectively, were used. As shown in Fig. 2E and F, after co-staining of the HepG2 cells with AIEgens and MitoTracker, a significant overlapped fluorescence between the two was observed, with Pearson's correlations (*R*) being 0.88, 0.87 and 0.88 for LOCK-0, LOCK-1 and LOCK-2, respectively. This suggests good mitochondrial-targeting capacity of the AIEgens bearing the cationic triphenylphosphine group. However, the results of co-staining experiments with AIEgens and LysoTracker showed a much lower correlation (Fig. 2G and S11†).

**Fig. 3:**
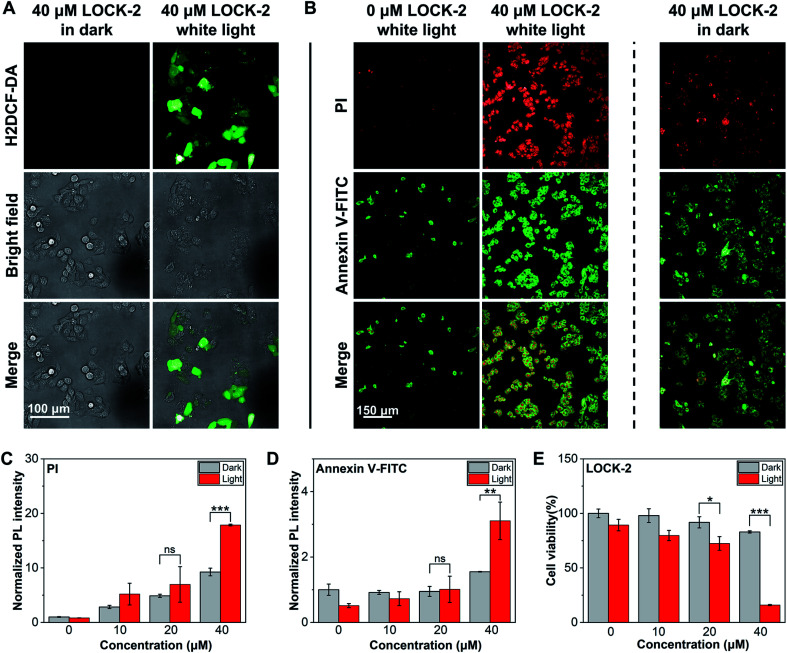
(A) Fluorescence imaging of HepG2 cells with LOCK-2 stained with H2DCF-DA with and without white light irradiation by confocal laser-scanning microscopy. (B) Fluorescence imaging of HepG2 cells with LOCK-2 stained with PI, Annexin V-FITC with and without white light irradiation by confocal laser-scanning microscopy. Fluorescence quantification of HepG2 cells with LOCK-2 stained with PI (C) and Annexin V-FITC (D) with and without white light irradiation. (E) Cell viability of HepG2 cells after treatment with various concentrations of LOCK-2 with and without white light irradiation. H2DCF-DA concentration: 10 μM. Annexin V-FITC/PI concentration: 1 : 100 of stock solution. MTS/PMS: 20 : 1, Promega Corp, 20 μL per well. Excitation source: H2DCF-DA: 488 nm; PI: 561 nm; Annexin V-FITC: 488 nm. The emission filter: H2DCF-DA: 500–550 nm; PI: 570–630 nm; Annexin V-FITC: 500–550 nm. Irradiation time: 2 h. White light: 22.7 mW cm^−2^. ***P < 0.001, **P < 0.01, *P < 0.05, ns: not significant.

Next, we analyzed the PDT effect of the AIEgens for HepG2 cells. LOCK-2 with the best ROS yield as determined in solution-based tests was chosen. First, the *in situ* ROS generation of the probes was tested using H2DCF-DA as the indicator (Fig. 3A). While minimal fluorescence was observed after incubation of HepG2 cells with 40 μM LOCK-2 in the dark, strong green fluorescence of DCF occurred with white light irradiation (22.7 mW cm^−2^) for 2 h. This indicates the intracellular production of ROS by LOCK-2 after light irradiation. Then, PDT-induced apoptosis was evaluated using commercial Annexin V-FITC/PI (Propidium Iodide) double staining assay kit. Early apoptosis of cells is indicated by the green fluorescence of Annexin V-FITC, and late cell apoptosis or necrosis is indicated by the red fluorescence of PI. When HepG2 cells were incubated with LOCK-2 and then irradiated with white light (22.7 mW cm^−2^) for 2 h, the fluorescence intensities of Annexin V-FITC and PI were found to be much stronger than those without light irradiation; the PDT effect was also found to be concentration-dependent (Fig. 3B–D and S12 and S13†). Subsequently, the cell viability was measured using an MTS assay. As illustrated in Fig. 3E and S14,† the cell viability of HepG2 cells after incubation with increasing concentrations of LOCK-2 was not reduced in the dark, suggesting its low dark toxicity. However, when light was applied, a significantly reduced cell viability (15%) was observed when 40 μM of the AIEgen was used. Moreover, the *in vitro* PDT efficiency of LOCK-2 was further evaluated with commercial PSs, Ce6 (Chlorin e6) and RB. As shown in Fig. S15,†LOCK-2 exhibited high phototoxicity similar to that of Ce-6 and RB at the concentration of 40 μM, indicating the great potential of LOCK-2 as a PDT agent for cancer therapy.

## Conclusions

Three water-soluble AIEgens for FI-guided PDT were developed. LOCK-2 exhibited attenuated AIE and TICT effects resulting in improved QY and ROS yields. We ascribed this observation to the suppressed intramolecular motions of LOCK-2 facilitating the conversion of excited photonic energy into fluorescence emission and triplet excitons. Due to good water-solubility and electrostatic interactions with MMP, mitochondrial-targeting FI of HepG2 cells was achieved using a wash-free protocol. In addition, LOCK-2 exhibited concentration-dependent PDT effects in HepG2 cells with minimal cytotoxicity in the dark. We expect that our simple molecular engineering strategies will help guide the development of AIEgens with improved QY and ROS yields for FI-guided PDT in practical applications.

## Data availability

Methods, materials, characterization, theoretical results and other supporting information are available in the ESI.†

## Author contributions

F.-Z. X., X.-P. H. and C.-Y. W. conceived the project and designed the experiments. F.-Z. X., L. Z. and H.-H. H performed the experimental work. J.-W. Z. performed the theoretical calculations. L. Z. and Y. Z. analysed and interpreted the data. The manuscript was written by F.-Z. X., L. Z. and H.-H. H. and revised by J.-W. Z., J. L., T. D. J., X.-P. H. and C.-Y. W. All of the authors contributed to the discussion of the results.

## Conflicts of interest

The authors declare no competing financial interests.

## Supplementary Material

SC-013-D2SC00889K-s001
