# Supplementary material for: Molecularly engineered AIEgens with enhanced quantum and singlet-oxygen yield for mitochondria-targeted imaging and photodynamic therapy
Source: Chem Sci. 2022 Aug 3;13(32):9373–80. doi: 10.1039/d2sc00889k (PMC9384827; doi:10.1039/d2sc00889k)
Supplement: SC-013-D2SC00889K-s001 [file SC-013-D2SC00889K-s001.pdf]

Electronic Supplementary Material (ESI) for Chemical Science.
This journal is © The Royal Society of Chemistry 2022

Supporting Information

Molecularly Engineered AIEgens with Enhanced Quantum and Singlet-Oxygen Yield for Mitochondrion-Targeted Cell Imaging and Photodynamic Therapy

Fang-Zhou Xu,‡[a] Ling Zhu,‡[a] Hai-Hao Han,‡[a] Jian-Wei Zou,*[b] Yi Zang,[c,d] Jia Li, *[c,d] Tony D. James, *[e,f] Xiao-Peng He, *[a] and Cheng-Yun Wang*[a]

[a] Key Laboratory for Advanced Materials and Joint International Research Laboratory of Precision Chemistry and Molecular Engineering, Feringa Nobel Prize Scientist Joint Research Center, Frontiers Center for Materiobiology and Dynamic Chemistry, School of Chemistry and Molecular Engineering, East China University of Science and Technology, 130 Meilong Rd., Shanghai 200237, China. E-mail: xphe@ecust.edu.cn; cywang@ecust.edu.cn

[b] NingboTech University, Ningbo 315100, Zhejiang, PR China. E-mail: jwzou@nit.net.cn

[c] National Center for Drug Screening, State Key Laboratory of Drug Research, Shanghai Institute of Materia Medica, Chinese Academy of Sciences, Shanghai 201203, China. E-mail: jli@simm.ac.cn

[d] University of Chinese Academy of Sciences, No. 19A Yuquan Road, Beijing 100049, P. R. China

[e] Department of Chemistry, University of Bath, Bath BA2 7AY, UK. E-mail: t.d.james@bath.ac.uk

[f] School of Chemistry and Chemical Engineering, Henan Normal University, Xinxiang 453007, China

‡ Equal contribution

Table of Contents

Scheme S1 Synthetic routes of **LOCK-0**, **LOCK-1**, and **LOCK-2**. S10
Fig. S1 UV-visible absorption spectra of **LOCK-0**, **LOCK-1** and **LOCK-2** in aqueous solution......S10
Fig. S2 PL spectra of **LOCK-0**, **LOCK-1** and **LOCK-2** in THF/H_2O solution......S10
Fig. S3 Solvent effect experiments of **LOCK-0**, **LOCK-1** and **LOCK-2**......S11
Fig. S4 Dihedral angels (θ) of **LOCK-0**, **LOCK-1** and **LOCK-2** at ground state (S_0) and S_1 excited state......S11
Fig. S5 ROS detection by H2DCF for **LOCK-0**, **LOCK-1** and **LOCK-2**. S12
Fig. S6 Measurement and calculation of $^1O_2$ yields of **LOCK-0**, **LOCK-1** and **LOCK-2** under white light irradiation......S12
Fig. S7 Measurement and calculation of $^1O_2$ yields of **LOCK-0**, **LOCK-1** and **LOCK-2** under 480 or 520 nm laser irradiation.. ......S13
Fig. S8 Concentration-dependent fluorescence imaging by confocal laser-scanning of HepG2 cells with **LOCK-0**, **LOCK-1** and **LOCK-2** in washed and wash-free manner......S13
Fig. S9 Successive fluorescence imaging by confocal laser-scanning of HepG2 cells with **LOCK-0**, **LOCK-1** and **LOCK-2**.. ......S14
Fig. S10 Fluorescence quantification of HepG2 cells with **LOCK-0**, **LOCK-1** and **LOCK-2** in the photostability experiments. S14
Fig. S11 Fluorescence imaging of HepG2 cells with **LOCK-0**, **LOCK-1** and **LOCK-2** co-stained with LTG by confocal laser-scanning microscopy.. ......S15
Fig. S12 Fluorescence imaging of HepG2 cells with **LOCK-2** stained with PI, Annexin V-FITC with white light irradiation by confocal laser-scanning microscopy......S16
Fig. S13 Fluorescence imaging of HepG2 cells with **LOCK-2** stained with PI, Annexin V-FITC without white light irradiation by confocal laser-scanning microscopy......S16
Fig. S14 Cell viability of HepG2 cells after treatment of various concentrations of **LOCK-0**, **LOCK-1** and **LOCK-2** in dark for 24 h......S17
Fig. S15 Cell viability of HepG2 cells after treatment of various concentrations of **LOCK-0**, **LOCK-1**, **LOCK-2**, Ce6 and RB with and without white light irradiation......S17
Fig. S16 ^1H NMR spectrum of **compound 2** in CD_3OD......S18
Fig. S17 ^{13}C NMR spectrum of **compound 2** in CD_3OD. S18
Fig. S18 HRMS spectrum of **compound 2**. S19
Fig. S19 ^1H NMR spectrum of **compound 4** in DMSO-d_6. ......S19
Fig. S20 ^{13}C NMR spectrum of **compound 4** in DMSO-d_6. ......S20
Fig. S21 HRMS spectrum of **compound 4**. S20

Fig. S22 ^{1}H NMR spectrum of **compound 5** in DMSO-d_6. ....S21
Fig. S23 ^{13}C NMR spectrum of **compound 5** in DMSO-d_6. ....S21
Fig. S24 HRMS spectrum of **compound 5**. S22
Fig. S25 ^{1}H NMR spectrum of **compound 6** in DMSO-d_6. ....S22
Fig. S26 ^{13}C NMR spectrum of **compound 6** in DMSO-d_6. ....S23
Fig. S27 HRMS spectrum of **compound 6**. S23
Fig. S28 ^{1}H NMR spectrum of **compound 7** in DMSO-d_6. ....S24
Fig. S29 ^{13}C NMR spectrum of **compound 7** in DMSO-d_6. ....S24
Fig. S30 HRMS spectrum of **compound 7**. S25
Fig. S31 ^{1}H NMR spectrum of **LOCK-0** in DMSO-d_6. ....S25
Fig. S32 ^{13}C NMR spectrum of **LOCK-0** in DMSO-d_6. ....S26
Fig. S33 HRMS spectrum of **LOCK-0**. S26
Fig. S34 ^{1}H NMR spectrum of **LOCK-1** in DMSO-d_6. ....S27
Fig. S35 ^{13}C NMR spectrum of **LOCK-1** in DMSO-d_6. ....S27
Fig. S36 HRMS spectrum of **LOCK-1**. S28
Fig. S37 ^{1}H NMR spectrum of **LOCK-2** in DMSO-d_6. ....S28
Fig. S38 ^{13}C NMR spectrum of **LOCK-2** in DMSO-d_6. ....S29
Fig. S39 HRMS spectrum of **LOCK-2**. S29
Table S1 Energy levels and energy gaps of HOMO and LUMO for **LOCK-0**, **LOCK-1** and **LOCK-2**.. ....S30
Table S2 Electronic excitation energy levels of singlet and triplet states for **LOCK-0**........S30
Table S3 Electronic excitation energy levels of singlet and triplet states for **LOCK-1**........S30
Table S4 Electronic excitation energy levels of singlet and triplet states for **LOCK-2**........S31

Materials and methods

All chemicals are commercially available without further purification. The NMR spectra were recorded with a Bruker AM 400 spectrometer or an Ascend 600 spectrometer. High resolution mass spectra were recorded with a Waters LCT Premier XE spectrometer. UV-vis spectra were recorded with an Agilent Cary 60 spectrophotometer. Fluorescence spectra were recorded with an Agilent Cary Eclipse fluorescence spectrophotometer. Absolute QY was measured by an Edinburgh FLS980 fluorescence spectrophotometer with a standard integrating sphere. EPR spectra were recorded with a Brucker EMX-8/2.7 electro-spin resonance spectrometer. Confocal cell images in the colocalization experiments were recorded with a Leica TCS SP8 laser scanning confocal microscopy. Other confocal cell images were recorded with Opera Phenix high content imaging system and quantified by the Columbus image data analysis system (Perkinelmer, US). Absorbance of the solutions in MTS assay was measured by a M5 microplate reader (Molecular Device, USA).

Synthesis of LOCK-0, LOCK-1, and LOCK-2

Compounds 1 and **3** were prepared according to previous literature reports.[1, 2]

Compound 2. A solution of **compound 1** (1.91 g, 4.0 mmol) and 4-methyl-quinoline (0.57 g, 4.0 mmol) in acetonitrile (30 mL) was refluxed under nitrogen for 48 h. After cooling to room temperature, the solvent was removed by evaporation under reduced pressure and then dissolved in minimal amount of methanol. The methanol solution was dropped into ether (80 mL) to yield a pale pink precipitate. After decantation, the precipitate was carefully washed with ethyl acetate (30 mL × 3) to afford a white solid (2.24 g, 90% of yield). ^{1}H NMR (400 MHz, CD_3OD, ppm): δ 9.30 (d, J = 6.1 Hz, 1 H), 8.58–8.55 (m, 2 H), 8.27–8.23 (m, 1 H), 8.05 (t, J = 7.2 Hz, 1 H), 7.94 (d, J = 6.1 Hz, 1 H), 7.92–7.83 (m, 7 H), 7.81 (d, J = 1.4 Hz, 2 H), 7.76 (td, J = 7.8, 3.6 Hz, 6 H), 5.09 (t, J = 7.6 Hz, 2 H), 3.60 (td, J = 15.0, 2.8 Hz, 2 H), 3.05 (s, 3 H), 2.38–2.31 (m, 2 H), 1.96–1.86 (m, 2 H); ^{13}C NMR (101 MHz, CD_3OD, ppm): δ 161.05, 149.23, 138.73, 136.41, 135.00, 131.73, 131.59, 130.98, 128.43, 128.27, 124.01, 123.88, 120.55, 120.39, 120.12, 119.26, 57.78, 31.86, 31.69, 22.77, 22.25, 20.87, 20.47. Mass spectrometry (ESI-MS, *m/z*): $[M\text{-}2Br]^{2+}$ calcd. for $C_{32}H_{32}NP^{2+}$, 230.6134; found, 230.6077.

Compound 4. A solution of diphenylamine (784 mg, 4.7 mmol), 5-bromo-benzothiophene (1.00 g, 4.7 mmol), ^{t}BuONa (496 mg, 5.17 mmol), $Pd_2(dba)_3$ (215 mg, 0.24 mmol) in toluene (50 mL) was refluxed under nitrogen overnight. After cooling to room temperature, the solution

was washed with water (20 mL × 3) and the solvent was evaporated under reduced pressure. The residue was purified with a silica gel column using a PE and DCM mixture (10:1 v/v) as the eluting solvent to afford a white powder (1.27 g, 90% of yield). ^{1}H NMR (400 MHz, DMSO-d_6, ppm): δ 7.92 (d, J = 8.7 Hz, 1 H), 7.75 (d, J = 5.4 Hz, 1 H), 7.52 (d, J = 2.1 Hz, 1 H), 7.35 (d, J = 5.4 Hz, 1 H), 7.28 (t, J = 7.9 Hz, 4 H), 7.08 (dd, J = 8.6, 2.1 Hz, 1 H), 7.03–6.99 (m, 6 H); ^{13}C NMR (101 MHz, DMSO-d_6, ppm): δ 147.60, 144.26, 140.81, 134.31, 129.45, 128.46, 123.92, 123.56, 123.24, 123.15, 122.48, 119.20. Mass spectrometry (ESI-MS, *m/z*): $[M+H]^+$ calcd. for $C_{20}H_{16}NS^+$, 302.1003; found, 302.1000.

Compound 5. DMF (364 mg, 4.98 mmol) in a flask was placed in an ice water bath. $POCl_3$ (762 mg, 4.98 mmol) was added dropwise into the DMF with stirring during 30 min. Then a DCE (5 mL) solution of **compound 4** (500 mg, 1.66 mmol) was added dropwise into the mixture and the solution was heated to 80 °C and stirred for 4 h. After cooling to room temperature, the solvent was evaporated under reduced pressure. Water (100 mL) was poured into the residue and a white powder (508 mg, 93% of yield) was obtained through filtering. ^{1}H NMR (400 MHz, DMSO-d_6, ppm): δ 9.77 (s, 1 H), 8.06 (d, J = 8.6 Hz, 1 H), 7.82 (d, J = 5.4 Hz, 1 H), 7.74 (d, J = 2.1 Hz, 1 H), 7.72 (d, J = 8.8 Hz, 2 H), 7.45–7.40 (m, 3 H), 7.26–7.20 (m, 4 H), 6.90 (d, J = 8.7 Hz, 2 H); ^{13}C NMR (101 MHz, DMSO-d_6, ppm): δ 190.56, 153.04, 145.73, 142.36, 140.94, 136.45, 131.30, 129.95, 129.03, 128.37, 126.11, 121.62, 117.80. Mass spectrometry (ESI-MS, *m/z*): $[M+H]^+$ calcd. for $C_{21}H_{16}NOS^+$, 330.0953; found, 330.0956.

Compound 6. The synthesis of **compound 6** is similar to that of **compound 4**. ^{1}H NMR (400 MHz, DMSO-d_6, ppm): δ 8.30 (d, J = 8.5 Hz, 1 H), 8.27 (d, J = 7.7 Hz, 2 H), 8.16 (d, J = 1.9 Hz, 1 H), 7.96 (d, J = 5.4 Hz, 1 H), 7.60–7.57 (m, 2 H), 7.46–7.39 (m, 4 H), 7.30 (td, J = 7.0, 1.2 Hz, 2 H); ^{13}C NMR (101 MHz, DMSO-d_6, ppm): δ 140.77, 140.50, 138.26, 133.44, 129.52, 126.22, 124.16, 123.07, 122.62, 121.78, 120.51, 119.96, 109.61. Mass spectrometry (ESI-MS, *m/z*): $[M+H]^+$ calcd. for $C_{20}H_{14}NS^+$, 300.0847; found, 300.0848.

Compound 7. The synthesis of **compound 7** is similar to that of **compound 5**. ^{1}H NMR (400 MHz, DMSO-d_6, ppm): δ 10.11 (s, 1 H), 8.90 (d, J = 1.1 Hz, 1 H), 8.42 (d, J = 7.7 Hz, 1 H), 8.34 (d, J = 8.5 Hz, 1 H), 8.20 (d, J = 1.9 Hz, 1 H), 8.00–7.97 (m, 2 H), 7.62 (d, J = 2.3 Hz, 1 H), 7.60 (d, J = 2.1 Hz, 1 H), 7.54–7.50 (m, 2 H), 7.43–7.39 (m, 2 H); ^{13}C NMR (101 MHz, DMSO-d_6, ppm): δ 191.94, 143.98, 141.53, 140.76, 138.95, 132.56, 129.81, 129.30, 127.26, 124.16, 123.04, 122.83, 122.60, 121.16, 110.23. Mass spectrometry (ESI-MS, *m/z*): $[M+H]^+$ calcd. for $C_{21}H_{14}NOS^+$, 328.0796; found, 328.0790.

LOCK-0. A solution of **compound 2** (250 mg, 0.4 mmol), **compound 3** (130 mg, 0.4 mmol) and piperidine (1 drop) in EtOH (20 mL) was refluxed under nitrogen overnight. After cooling to room temperature, the solvent was removed by evaporation under reduced pressure. The residue was purified with a neutral aluminum oxide column using a DCM and methanol mixture (30:1 v/v) as the eluting solvent to afford a purple powder (191 mg, 50% of yield). ^{1}H NMR (400 MHz, DMSO-d_6, ppm): δ 9.45 (d, J = 6.5 Hz, 1 H), 8.96 (d, J = 8.9 Hz, 1 H), 8.56 (d, J = 8.9 Hz, 1 H), 8.50–8.44 (m, 2 H), 8.23 (t, J = 8.0 Hz, 1 H), 7.93–7.89 (m, 5 H), 7.82–7.78 (m, 13 H), 7.69 (d, J = 8.6 Hz, 2 H), 7.62 (d, J = 3.9 Hz, 1 H), 7.37 (t, J = 7.8 Hz, 4 H), 7.12 (t, J = 8.1 Hz, 6 H), 7.01 (d, J = 8.7 Hz, 2 H), 5.04 (t, J = 7.0 Hz, 2 H), 3.81 (t, J = 14.4 Hz, 2 H), 2.22–2.14 (m, 2 H), 1.73–1.67 (m, 2 H); ^{13}C NMR (151 MHz, DMSO-d_6, ppm): δ 152.75, 149.10, 148.45, 147.50, 147.01, 139.57, 138.30, 136.83, 135.41, 134.99, 134.18, 134.11, 130.80, 130.71, 130.25, 129.53, 127.37, 127.07, 126.77, 125.31, 125.01, 124.45, 122.61, 119.71, 119.15, 118.58, 117.88, 115.98, 55.57, 30.33, 30.21, 20.39, 20.05, 19.37. Mass spectrometry (ESI-MS, *m/z*): $[M-2Br]^{2+}$ calcd. for $C_{55}H_{47}N_2PS^{2+}$, 399.1596; found, 399.1593.

LOCK-1. The synthesis of **LOCK-1** is similar to that of **LOCK-0**. ^{1}H NMR (400 MHz, DMSO-d_6): δ 9.44 (d, J = 6.2 Hz, 1 H), 9.03 (d, J = 8.5 Hz, 1 H), 8.55 (d, J = 9.0 Hz, 1 H), 8.45 (d, J = 6.3 Hz, 1 H), 8.24–8.13 (m, 3 H), 8.05 (d, J = 8.4 Hz, 1 H), 8.00 (t, J = 7.8 Hz, 1 H), 7.93–7.87 (m, 6 H), 7.85–7.82 (m, 6 H), 7.78 (w, 6 H), 7.70 (s, 1 H), 7.43–7.39 (m, 3 H), 7.20 (d, J = 7.7 Hz, 4 H), 7.00 (d, J = 8.0 Hz, 2 H), 5.05 (t, J = 6.3 Hz, 2 H), 3.82 (t, J = 14.2 Hz, 2 H), 2.19–2.12 (m, 2 H), 1.72–1.67 (m, 2 H); ^{13}C NMR (151 MHz, DMSO-d_6): δ 153.57, 150.75, 147.44, 146.80, 144.00, 143.46, 141.41, 138.31, 136.29, 135.40, 134.17, 134.10, 131.25, 130.78, 130.70, 130.33, 129.43, 128.67, 126.92, 125.81, 125.03, 124.45, 123.73, 121.29, 120.49, 119.13, 118.56, 117.12, 115.82, 56.48, 30.30, 30.19, 20.40, 20.07, 19.37. Mass spectrometry (ESI-MS, *m/z*): $[M-2Br]^{2+}$ calcd. for $C_{53}H_{45}N_2PS^{2+}$, 386.1518; found, 386.1508.

LOCK-2. The synthesis of **LOCK-2** is similar to that of **LOCK-0**. ^{1}H NMR (400 MHz, DMSO-d_6): δ 9.50 (d, J = 6.6 Hz, 1 H), 9.19 (d, J = 8.7 Hz, 1 H), 9.10 (s, 1 H), 8.59 (d, J = 9.0 Hz, 1 H), 8.55 (d, J = 6.8 Hz, 1 H), 8.41 (d, J = 7.7 Hz, 1 H), 8.37 (d, J = 8.5 Hz, 1 H), 8.23 (d, J = 1.8 Hz, 1 H), 8.11 (d, J = 8.7 Hz, 1 H), 8.00 (d, J = 5.4 Hz, 1 H), 7.91 (t, J = 7.1 Hz, 4 H), 7.88–7.77 (m, 15 H), 7.65–7.63 (m, 2 H), 7.52 (d, J = 8.4 Hz, 2 H), 7.46–7.41 (m, 2 H), 5.08 (t, J = 6.9 Hz, 2 H), 3.83 (t, J = 14.6 Hz, 2 H), 2.22–2.15 (m, 2 H), 1.76–1.67 (m, 2 H); ^{13}C NMR (151 MHz, DMSO-d_6): δ 153.75, 147.58, 145.40, 142.66, 141.87, 141.30, 139.26, 138.39, 135.43, 134.17, 134.10, 130.80, 130.72, 130.29, 129.46, 128.80, 128.44, 127.59, 127.04,

124.95, 124.70, 123.97, 123.47, 123.15, 122.36, 121.42, 119.12, 118.55, 117.41, 115.95, 110.81, 55.63, 30.30, 30.25, 20.39, 20.05, 19.40. Mass spectrometry (ESI-MS, *m/z*): $[M\text{-}2Br]^{2+}$ calcd. for $C_{53}H_{43}N_2PS^{2+}$, 385.1440; found, 385.1435.

Computational simulation. All theoretical calculation were performed using Gaussian 09 suite of program. Electronic excitation energies were calculated by TD-DFT at the CAM-B3LYP/6-31G(d) level in the treatment of solvent effects (water). In order to simplify the calculation, the number of carbon chains was set from 4 to 2.

ROS detection by H2DCF. H2DCF was obtained through hydrolysis of H2DCF-DA. PBS solutions of 10 μM AIEgens and 5 μM H2DCF were exposed to white light (400-700 nm, 10 mW cm^{-2}) irradiation, and then their fluorescence spectra at 523 nm were recorded on an Agilent Cary Eclipse fluorescence spectrophotometer.

$^1O_2$ yield detection. The absorption spectra of 5 μM AIEgens or RB aqueous solutions were recorded for the determination of A_{AIEgen} and A_{RB}, respectively. Then, 50 μM ABDA was added to the solutions, and the absorption spectra at 359, 378 and 399 nm were recorded with white light (400-700 nm, 10 mW cm^{-2}) or laser irradiation (520 nm for **LOCK-0**, **LOCK-1** and RB, 480 nm for **LOCK-2**, 7 mW cm^{-2}). The degradation rates for AIEgens and RB were calculated to be K_{AIEgen} and K_{RB}, respectively. Relative to the $^1O_2$ yield of RB as 0.75, the $^1O_2$ yields of AIEgens were calculated using the following equation.

$$\Phi_{AIEgen} = \Phi_{RB}\frac{K_{AIEgen}A_{RB}}{K_{RB}A_{AIEgen}}$$

where Φ are the $^1O_2$ yields of RB and AIEgens, *K* are the slopes of fitting line of RB and AIEgens, *A* are absorbance areas of RB and AIEgens, respectively.

$^1O_2$ detection by EPR. 0.5 μL TEMP ($^1O_2$ trapping agent) was added to 100 μL, 1.0 mM AIEgens aqueous solutions in dark. The EPR spectra of the resulting mixtures were recorded immediately with or without white light irradiation (400-700 nm, 10 mW cm^{-2}, 3 min) on a Brucker EMX-8/2.7 electro-spin resonance spectrometer.

Cell Culture. HepG2 cells (ATCC® HB-8065™) were cultured in a Dulbecco's Modified Eagle's Medium (DMEM, Gibco,12800082) supplemented with 10 % FBS (Gibco, 2025790) in a humidified atmosphere of 5 % CO_2 and 95 % air at 37 °C. Cells were split when reaching 90 % confluency.

Concentration-dependent washed and wash-free experiments. HepG2 cells (2.5×10^4 cells per well) were seeded in a black 96-well microplate with optically clear bottom (Greiner bio-one, 655090) and cultured in a humidified atmosphere of 5 % CO_2 and 95 % air at 37 °C overnight. Then, cells were incubated sequentially with Hoechst 33342 (5 $\mu g\cdot mL^{-1}$) for 5 min and AIEgens (0, 0.625, 1.25, 2.5, 5.0 μM) for 1 h, and rinsed with (for washed experiments) or without (for washed-free experiments) PBS before imaging. The fluorescence intensities were recorded using an Opera Phenix high content imaging system (Perkinelmer, US) and quantified and plotted by Columbus analysis system (Perkinelmer, US). **LOCK-2** channel excitation at 488 nm, emission at 570-630 nm; **LOCK-0** and **LOCK-1** channel excitation at 561 nm, emission at 650-760 nm; Hoechst 33342 channel excitation at 405 nm, emission at 410-480 nm.

Photostability. HepG2 cells were seeded in a black 96-well microplate with optically clear bottom (Greiner bio-one, 655090) in growth media supplemented with 10 % FBS and cultured overnight, and then treated with AIEgens (5.0 μM) for 1 h. After replacing the medium with fresh growth medium, cells were continuously imaged for 60 times (1 time/min) using an Opera Phenix high content imaging system (Perkinelmer, US) and quantified and plotted by Columbus analysis system (Perkinelmer, US).

Co-localization Experiment. HepG2 cells cultured in growth medium supplemented with 10 % FBS were added to a 24-well microplate (Corning, 3524), and cells were maintained in a humidified atmosphere of 5 % CO_2 and 95 % air at 37 °C overnight. Then the cells were incubated sequentially with AIEgens (5.0 μM) for 1 h, MitoTracker® Deep Red or LysoTracker® Deep Red (200 nM) for 40 min, and Hoechst 33342 (5 $\mu g\ mL^{-1}$) for 5 min. And cells were rinsed twice with warm PBS and fixed with 4% paraformaldehyde for 15 min at room temperature. After sealing, the fluorescence was detected and photographed using confocal laser scanning microscopy (Leica TCS SP8, Leica Microsystems, Wetzlar, Germany). **LOCK-2** channel excitation at 488 nm, emission at 570-650 nm; **LOCK-0** and **LOCK-1** channel excitation at 562 nm, emission at 600-800 nm; MitoTracker® Deep Red, LysoTracker® Deep Red channel excitation at 638 nm, emission at 648-710 nm; Hoechst 33342 channel excitation at 405 nm, emission at 415-487 nm.

ROS detection in cells. The intracellular ROS was further quantified using the Reactive Oxygen Species Assay Kit (MCE, HY-D0940). Briefly, HepG2 cells were seeded in a 96-well

plate (Greiner bio-one, 655090) in growth media supplemented with 10 % FBS and cultured overnight. And treated sequentially with AIEgens (40 μM) for 1 h and H2DCF-DA (10 μM) for 30 mins. Then, replace the medium with fresh one. Thereafter, the cells were irradiated with white light (22.7 mW·cm^{-2}) for 1 h. After rinsed with warm PBS, the fluorescence intensities were recorded by Opera Phenix high content imaging system and quantified and plotted by Columbus analysis system (Perkinelmer, US). DCFH-DA channel excitation at 488 nm, emission at 500-550 nm. The intracellular ROS level was directly proportional to the fluorescence intensity.

Annexin V-FITC/PI co-stain experiments. HepG2 cells (2.5×10^4 cells per well) were seeded in a black 96-well microplate with optically clear bottom (Greiner bio-one, 655090) and cultured overnight. Then, cells were treated with AIEgens at different concentrations (0, 10, 20, 40 μM) for 1 h. Then replace medium with fresh one. Thereafter, the cells were irradiated using white light (22.7 mW cm^{-2}) for 2 h, and then incubated with the mixture of Annexin V-FITC and PI solution (1:100 diluted by serum-free medium) for 30 min, and then washed by PBS. The fluorescence intensities were recorded using an Opera Phenix high content imaging system (Perkinelmer, US) and quantified and plotted by Columbus analysis system (Perkinelmer, US). Annexin V-FITC Channel excitation at 488 nm, emission at 500-550 nm; PI channel excitation at 561 nm, emission at 570-630 nm.

Cell viability assay. HepG2 Cells were seeded onto clear bottom 96-well plates (Corning, 3599) in growth medium supplemented with 10 % FBS and cultured overnight. Then the cells were treated with AIEgens of different concentrations (0, 10, 20, and 40 μM) for 1 h. After replacing the culture medium to fresh one, HepG2 cells were irradiated with white light (22.7 mW·cm^{-2}) for 2 h. After further incubation of 24 h, a solution of MTS/PMS (20:1, Promega Corp, G5430) (20 μL per well) was added to each well. After incubation at 37 °C under 5 % CO_2 for 3 h, the absorbance of the solutions was measured at 490 nm using a M5 microplate reader (Molecular Device, USA).

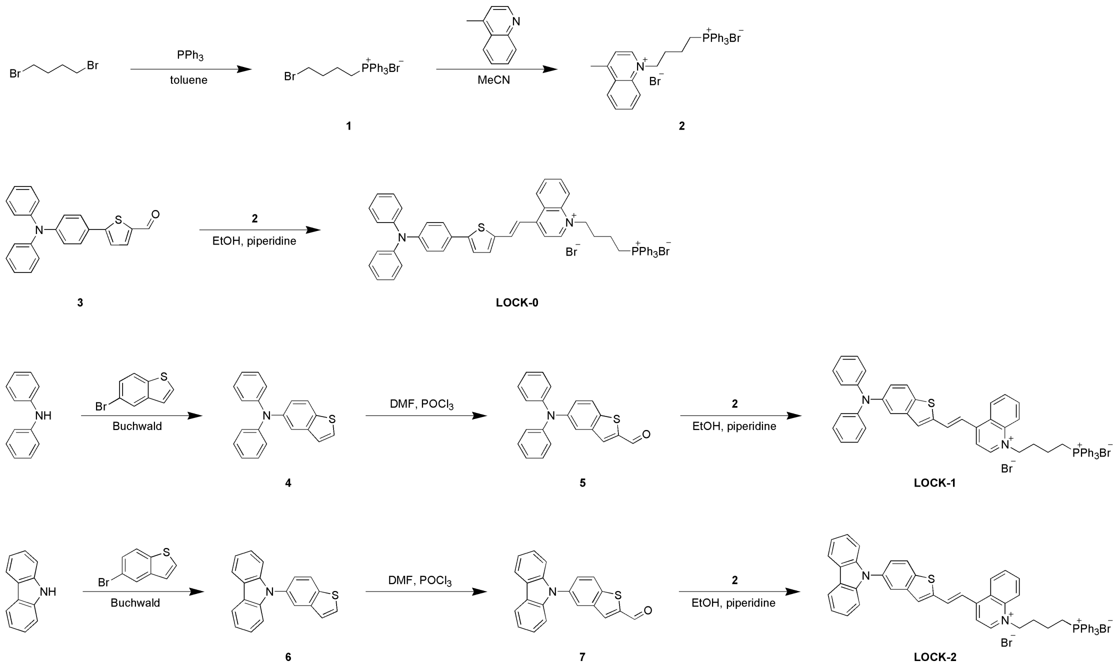

Scheme S1 Synthetic routes of **LOCK-0**, **LOCK-1**, and **LOCK-2**.

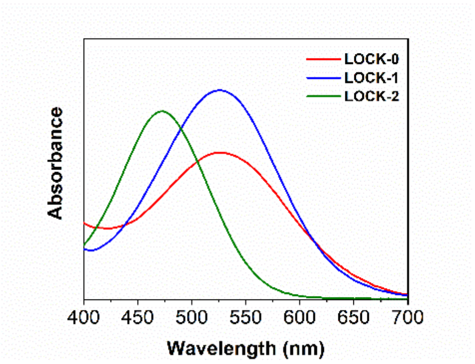

Fig. S1 UV-visible absorption spectra of **LOCK-0**, **LOCK-1** and **LOCK-2** in aqueous solution. AIEgen concentration: 10 μM.

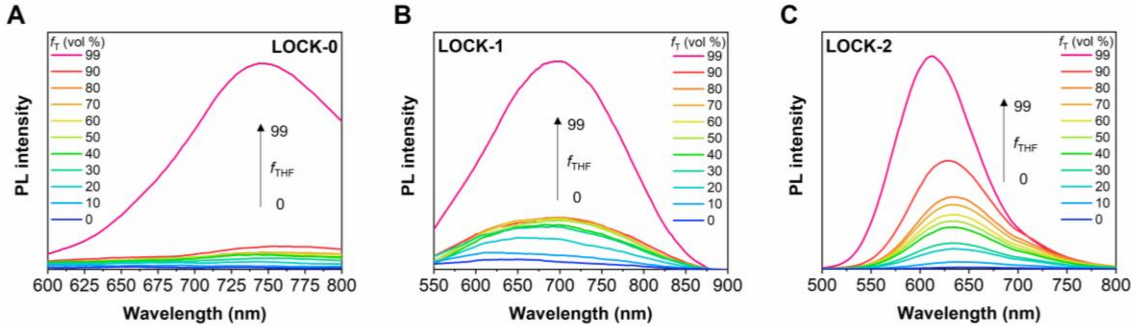

Fig. S2 PL spectra of **LOCK-0** (A), **LOCK-1** (B) and **LOCK-2** (C) in THF/H_2O solution.

AIEgen concentration: 10 μM. λ_{ex}: **LOCK-0** and **LOCK-1**: 523 nm; **LOCK-2**: 473 nm.

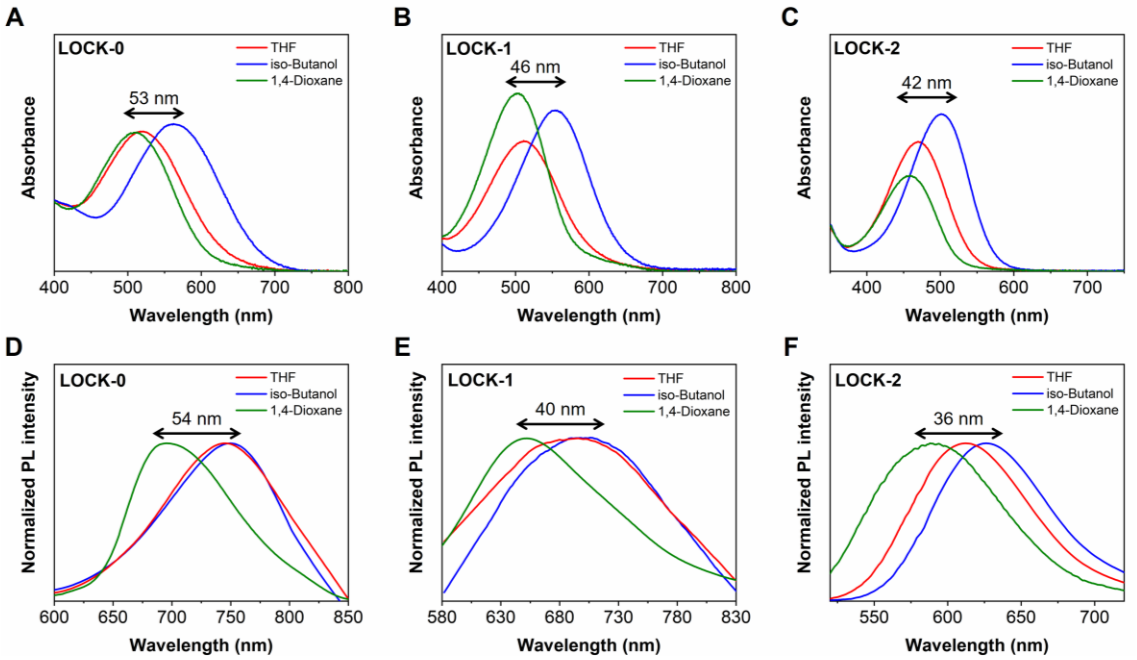

Fig. S3 Solvent effect experiments of **LOCK-0**, **LOCK-1** and **LOCK-2**. Absorption spectra of **LOCK-0** (A), **LOCK-1** (B) and **LOCK-2** (C) in THF, iso-butanol or 1,4-dioxane solution. AIEgen concentration: 10 μM. Normalized PL spectra of **LOCK-0** (D), **LOCK-1** (E) and **LOCK-2** (F) in THF, iso-butanol or 1,4-dioxane solution. AIEgen concentration: 10 μM.

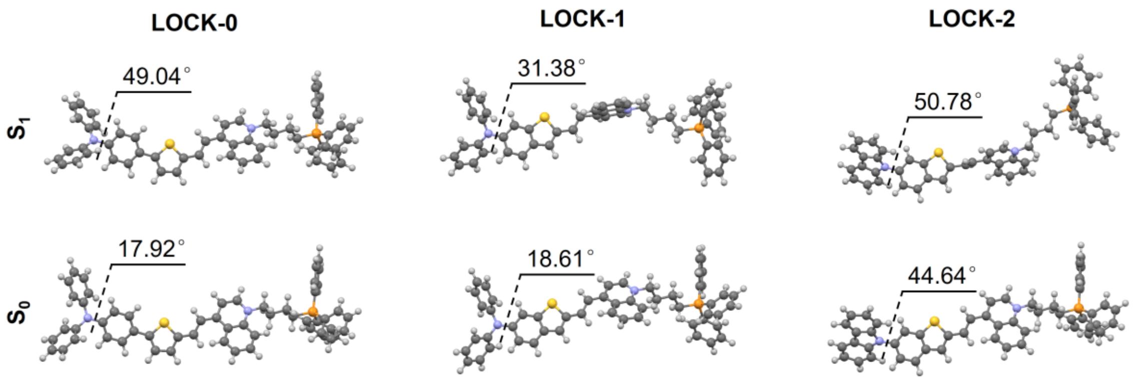

Fig. S4 Dihedral angels (θ) of **LOCK-0**, **LOCK-1** and **LOCK-2** at ground state (S_0) and S_1 excited state.

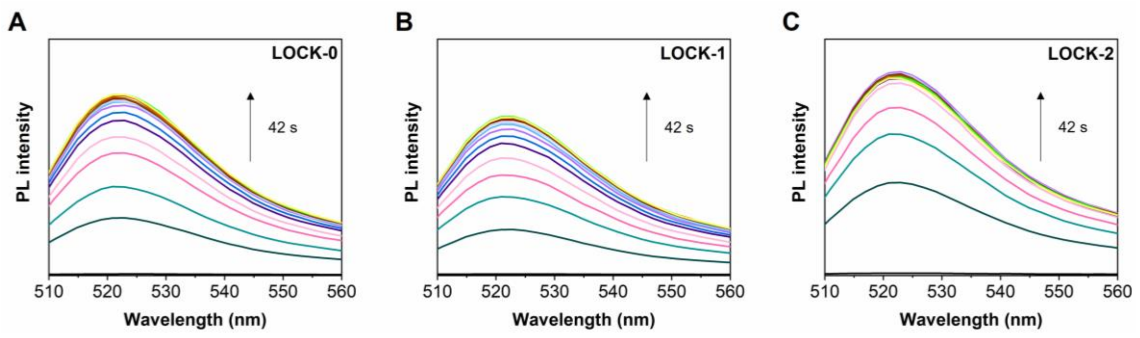

Fig. S5 ROS detection by H2DCF for **LOCK-0**, **LOCK-1** and **LOCK-2**. PL spectra of H2DCF with **LOCK-0** (A), **LOCK-1** (B) and **LOCK-2** (C) under different time of white light irradiation. AIEgen concentration: 10 μM. H2DCF concentration: 5 μM. White light: 400-700 nm, 10 mW cm^{-2}. λ_{ex}: 488 nm.

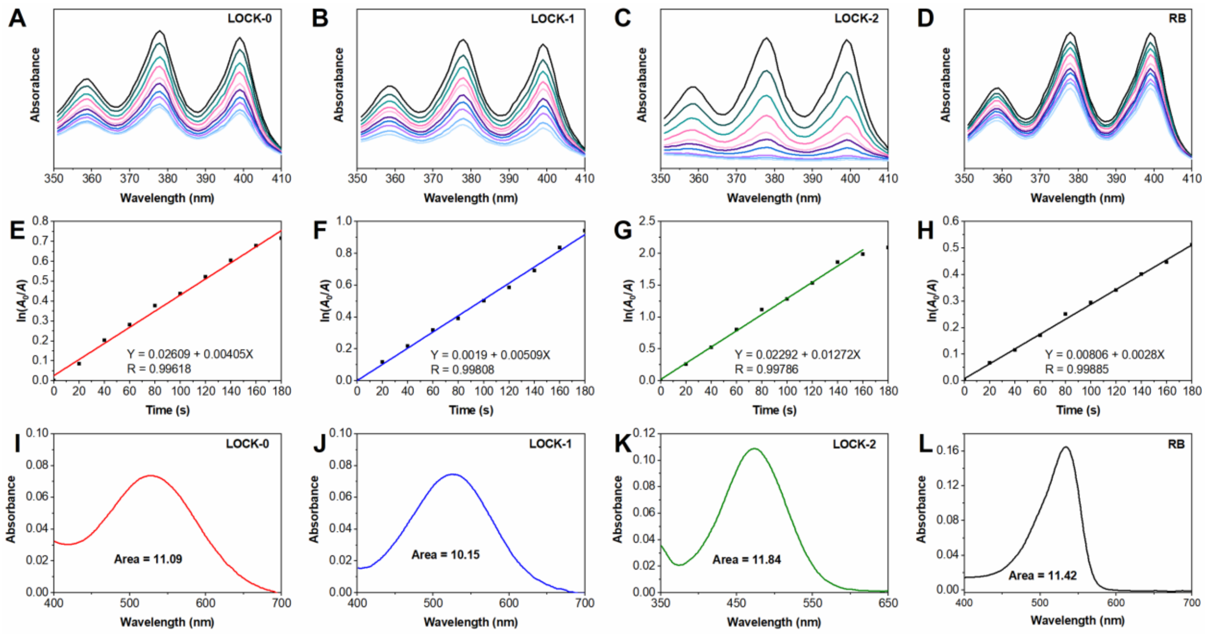

Fig. S6 Measurement and calculation of $^1O_2$ yields of **LOCK-0**, **LOCK-1** and **LOCK-2** under white light irradiation. UV-visible absorption spectra of ABDA aqueous solution with **LOCK-0** (A), **LOCK-1** (B), **LOCK-2** (C) and RB (D) under different time of white light irradiation. Linear fitting of absorbance area of ABDA at 359, 378 and 399 nm with **LOCK-0** (E), **LOCK-1** (F), **LOCK-2** (G) and RB (H). UV-visible absorption spectra of **LOCK-0** (I), **LOCK-1** (J), **LOCK-2** (K) and RB (L). AIEgen concentration: 5 μM. ABDA concentration: 50 μM. White light: 400-700 nm, 10 mW cm^{-2}.

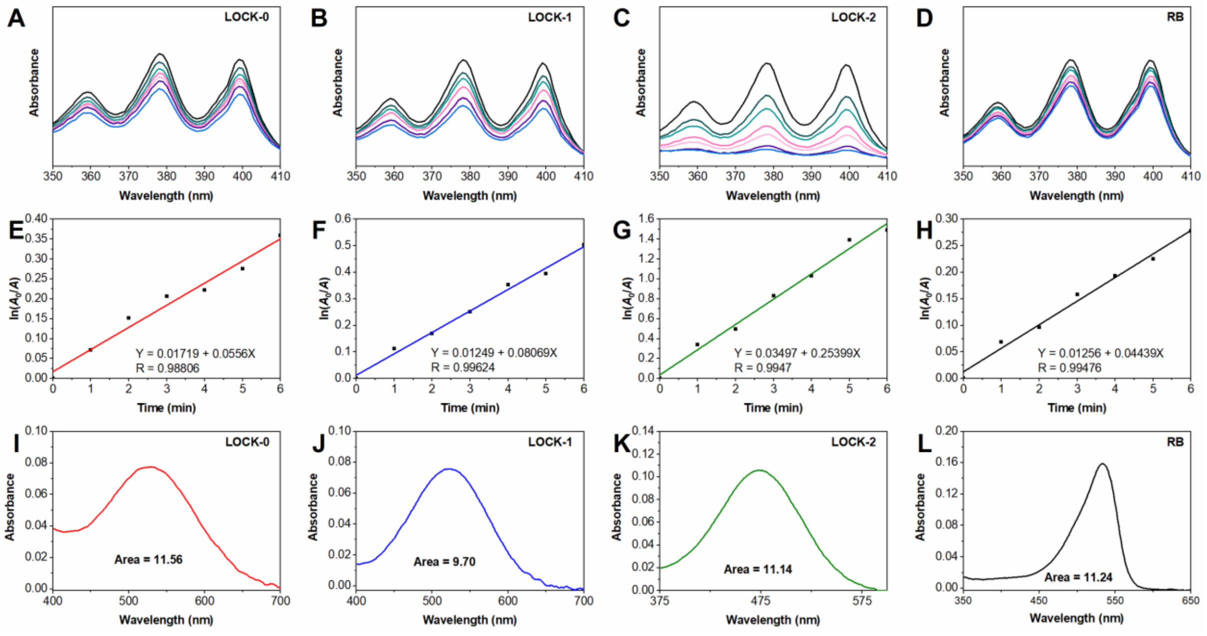

Fig. S7 Measurement and calculation of $^{1}O_2$ yields of **LOCK-0**, **LOCK-1** and **LOCK-2** under 480 or 520 nm laser irradiation. UV-visible absorption spectra of ABDA aqueous solution with **LOCK-0** (A), **LOCK-1** (B), **LOCK-2** (C) and RB (D) under 480 or 520 nm laser irradiation. Linear fitting of absorbance area of ABDA at 359, 378 and 399 nm with **LOCK-0** (E), **LOCK-1** (F), **LOCK-2** (G) and RB (H). UV-visible absorption spectra of **LOCK-0** (I), **LOCK-1** (J), **LOCK-2** (K) and RB (L). AIEgen concentration: 5 μM. ABDA concentration: 50 μM. Laser: 520 nm laser for **LOCK-0**, **LOCK-1** and RB, 480 nm laser for **LOCK-2**, 7 mW cm^{-2}.

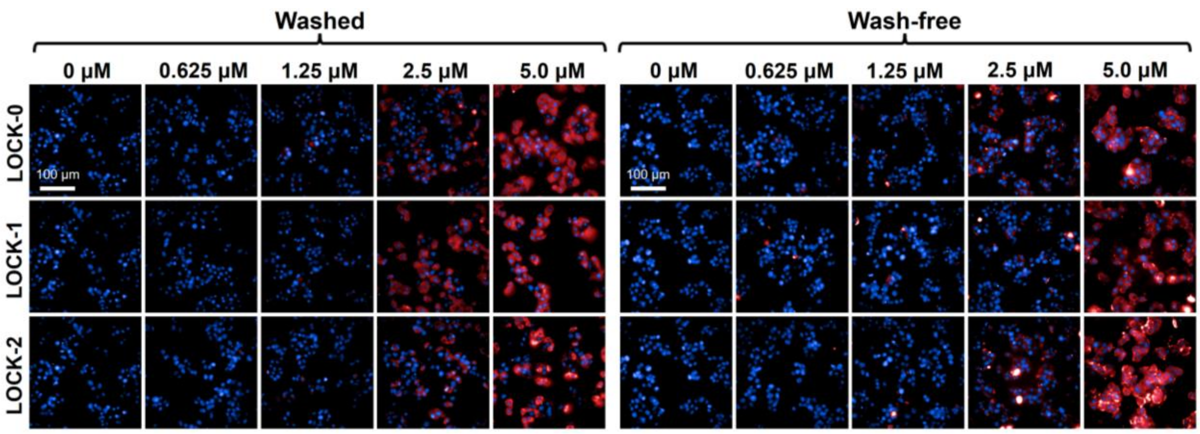

Fig. S8 Concentration-dependent fluorescence imaging by confocal laser-scanning of HepG2 cells with **LOCK-0**, **LOCK-1** and **LOCK-2** in washed and wash-free manner. Hoechst 33342 concentration: 5 μg mL^{-1}. The scale bar of all images: 100 μm. Excitation source: Hoechst 33342: 405 nm; **LOCK-0**: 561 nm; **LOCK-1**: 561 nm; **LOCK-2**: 488 nm. The emission filter:

Hoechst 33342: 410-480 nm; **LOCK-0**: 650-760 nm; **LOCK-1**: 650-760 nm; **LOCK-2**: 570-630 nm.

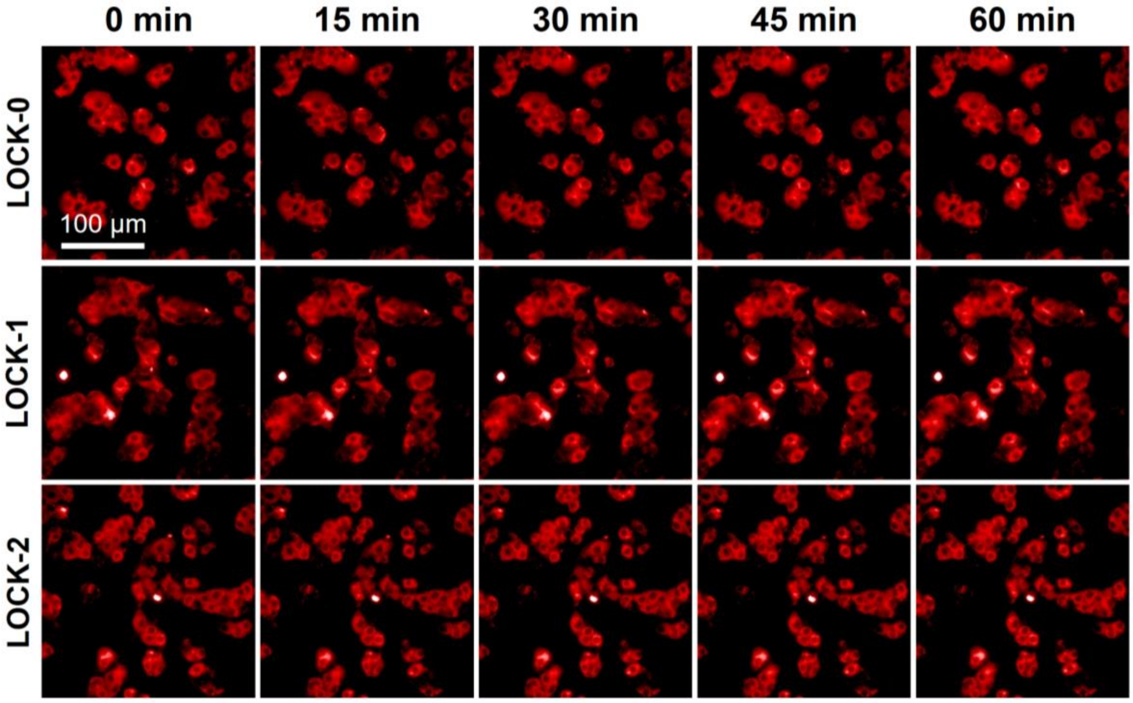

Fig. S9 Successive fluorescence imaging by confocal laser-scanning of HepG2 cells with **LOCK-0**, **LOCK-1** and **LOCK-2**. AIEgen concentration: 5 μM. Scanning: 60 times, 1 time/min. The scale bar of all images: 100 μm. Excitation source: **LOCK-0**: 561 nm; **LOCK-1**: 561 nm; **LOCK-2**: 488 nm. The emission filter: **LOCK-0**: 650-760 nm; **LOCK-1**: 650-760 nm; **LOCK-2**: 570-630 nm.

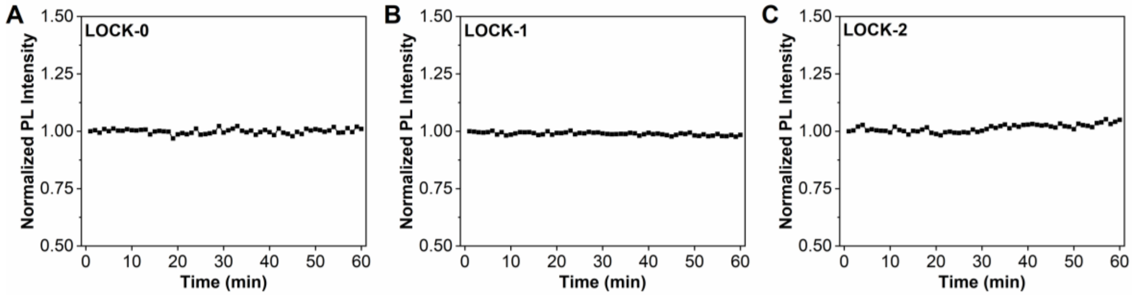

Fig. S10 Fluorescence quantification of HepG2 cells treated with **LOCK-0**, **LOCK-1** and **LOCK-2** in the photostability experiments.

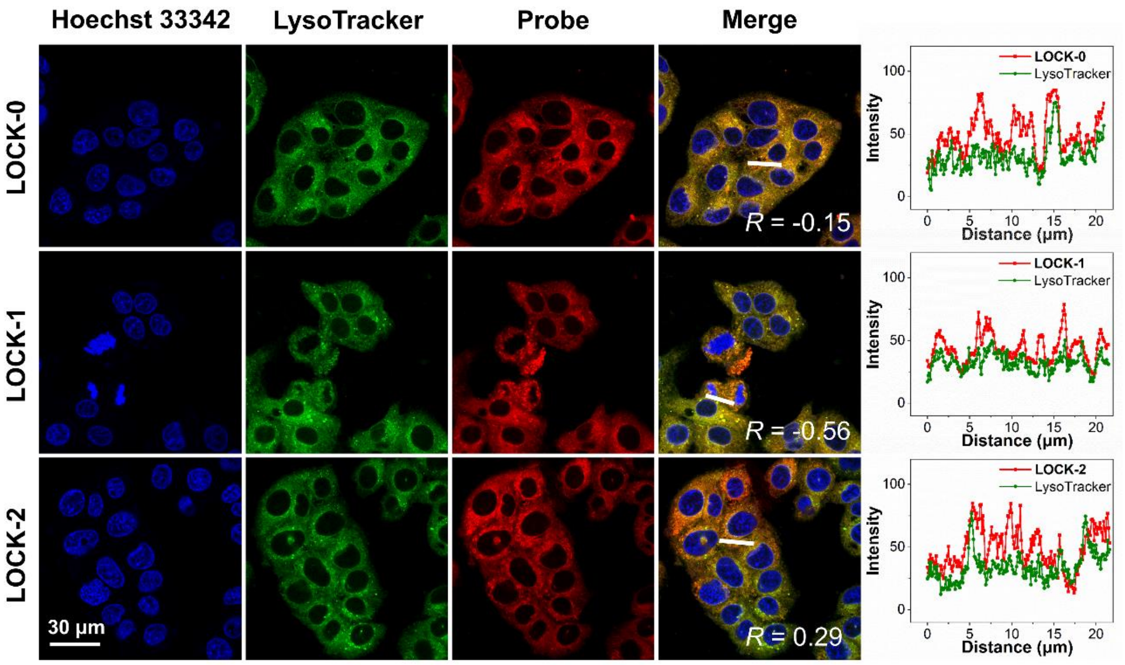

Fig. S11 Fluorescence imaging and quantification of a selected section of HepG2 cells with **LOCK-0**, **LOCK-1** and **LOCK-2** co-stained with LysoTracker® Deep Red by confocal laser-scanning microscopy. Hoechst 33342 concentration: 5 µg mL^{-1}. LysoTracker® Deep Red concentration: 200 nM. AIEgen concentration: 5 µM. The scale bar of all images: 30 µm. Excitation source: Hoechst 33342: 405 nm; LysoTracker® Deep Red: 638 nm; **LOCK-0**: 562 nm; **LOCK-1**: 562 nm; **LOCK-2**: 488 nm. The emission filter: Hoechst 33342: 415-487 nm; LysoTracker® Deep Red: 648-710 nm; **LOCK-0**: 600-800 nm; **LOCK-1**: 600-800 nm; **LOCK-2**: 570-650 nm.

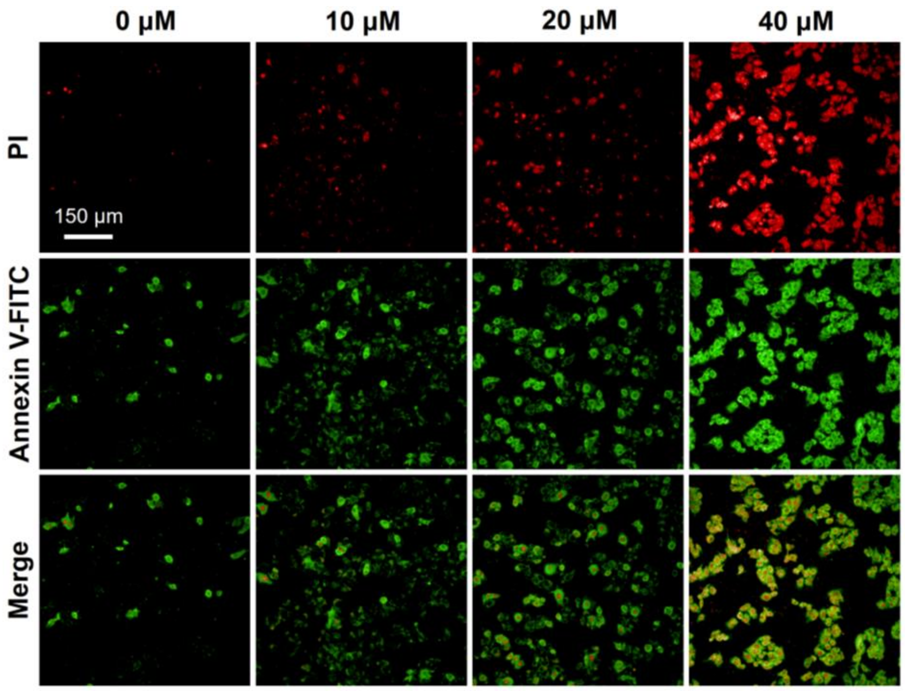

Fig. S12 Fluorescence imaging of HepG2 cells with **LOCK-2** stained with PI, Annexin V-FITC with white light irradiation by confocal laser-scanning microscopy. Annexin V-FITC/PI concentration: 1:100 of stock solution. Excitation source: PI: 561 nm; Annexin V-FITC: 488 nm. The emission filter: PI: 570-630 nm; Annexin V-FITC: 500-550 nm. Irradiation time: 2 h. White light: 22.7 mW cm^{-2}.

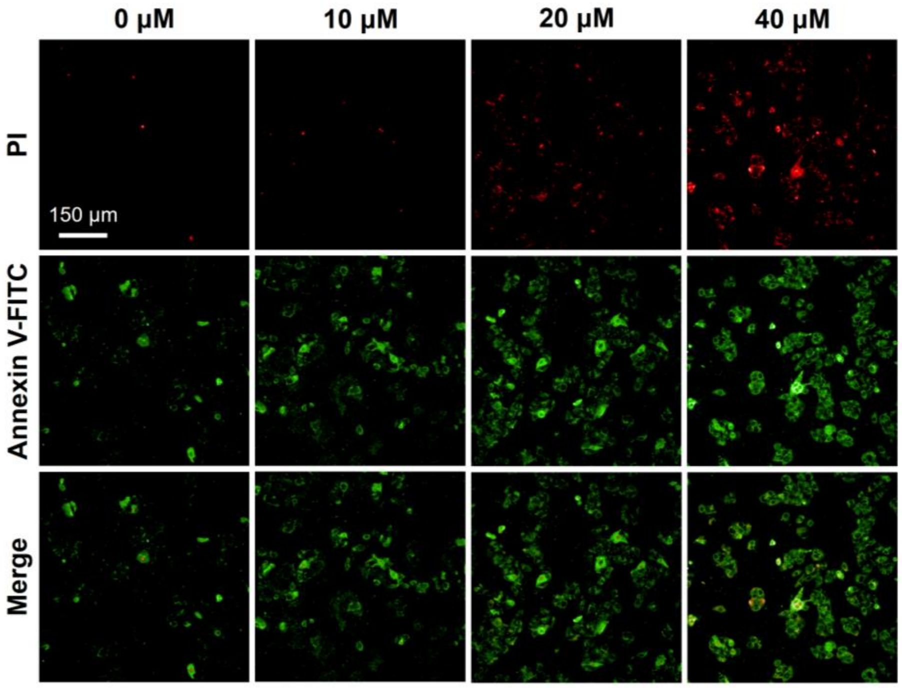

Fig. S13 Fluorescence imaging of HepG2 cells with **LOCK-2** stained with PI, Annexin V-

FITC without white light irradiation by confocal laser-scanning microscopy. Annexin V-FITC/PI concentration: 1:100 of stock solution. Excitation source: PI: 561 nm; Annexin V-FITC: 488 nm. The emission filter: PI: 570-630 nm; Annexin V-FITC: 500-550 nm. Irradiation time: 2 h. White light: 22.7 mW cm^{-2}.

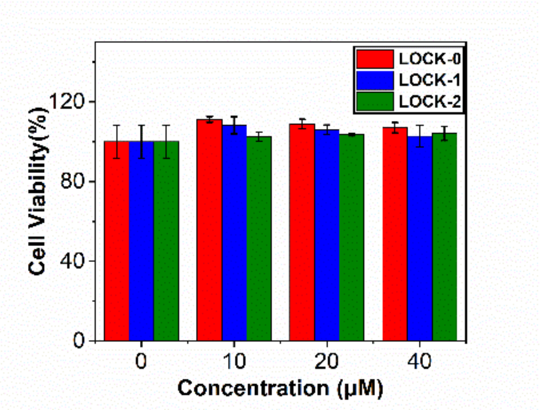

Fig. S14 Cell viability of HepG2 cells after treatment of various concentrations of **LOCK-0**, **LOCK-1** and **LOCK-2** in dark for 24 h.

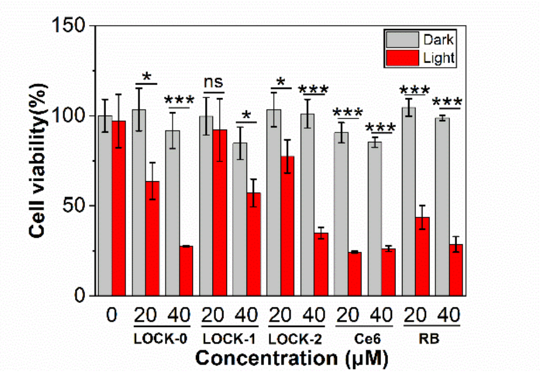

Fig. S15 Cell viability of HepG2 cells after treatment with various concentrations of **LOCK-0**, **LOCK-1**, **LOCK-2**, Ce6 and RB with and without white light irradiation. Irradiation time: 2 h. White light: 22.7 mW cm^{-2}. ***$P < 0.001$, *$P < 0.05$, ns: not significant.

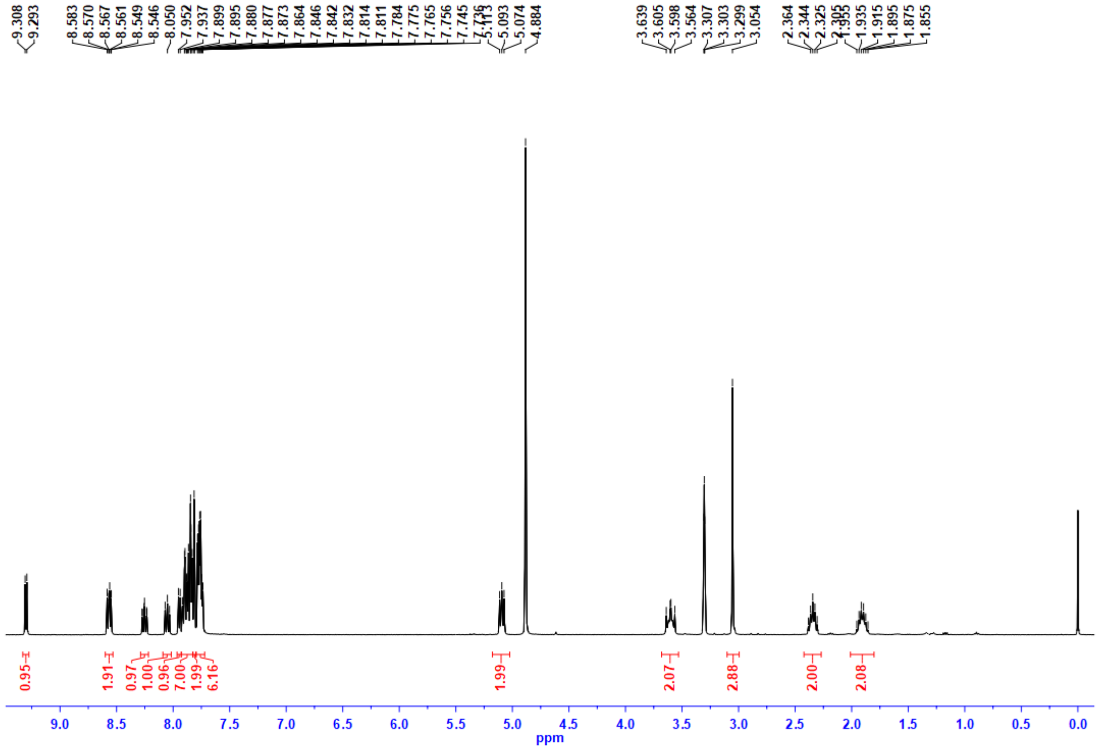

Fig. S16 ^{1}H NMR spectrum of **compound 2** in CD3OD.

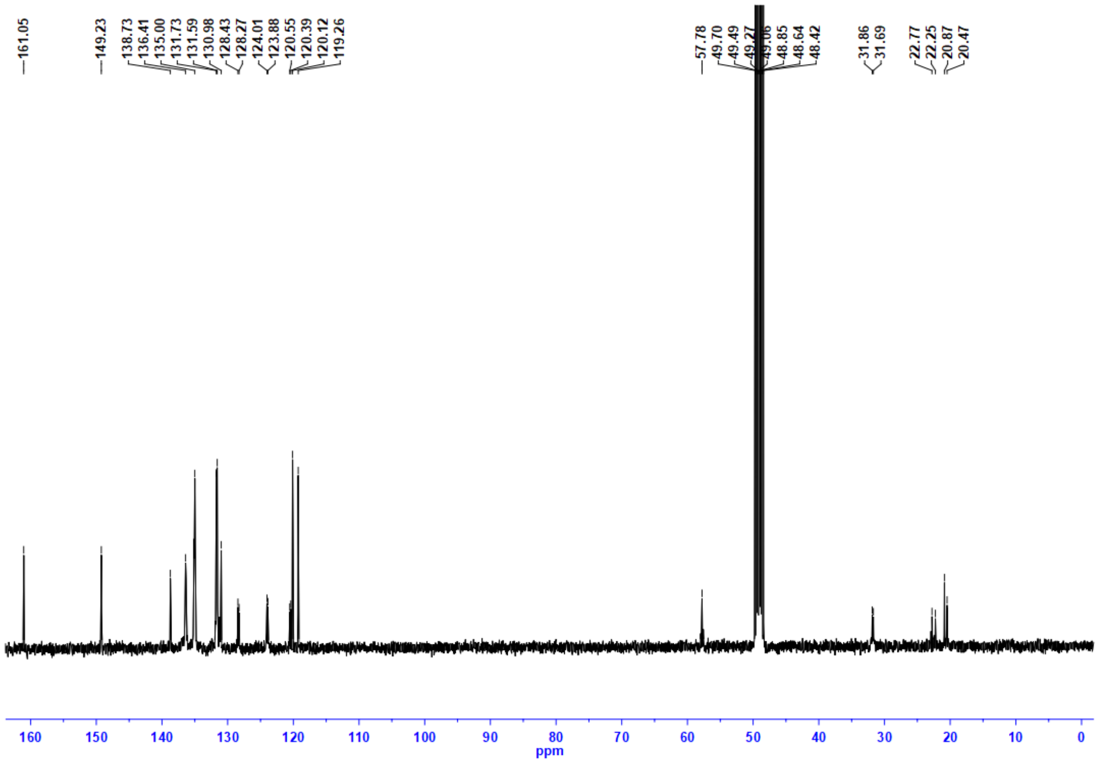

Fig. S17 ^{13}C NMR spectrum of **compound 2** in CD3OD.

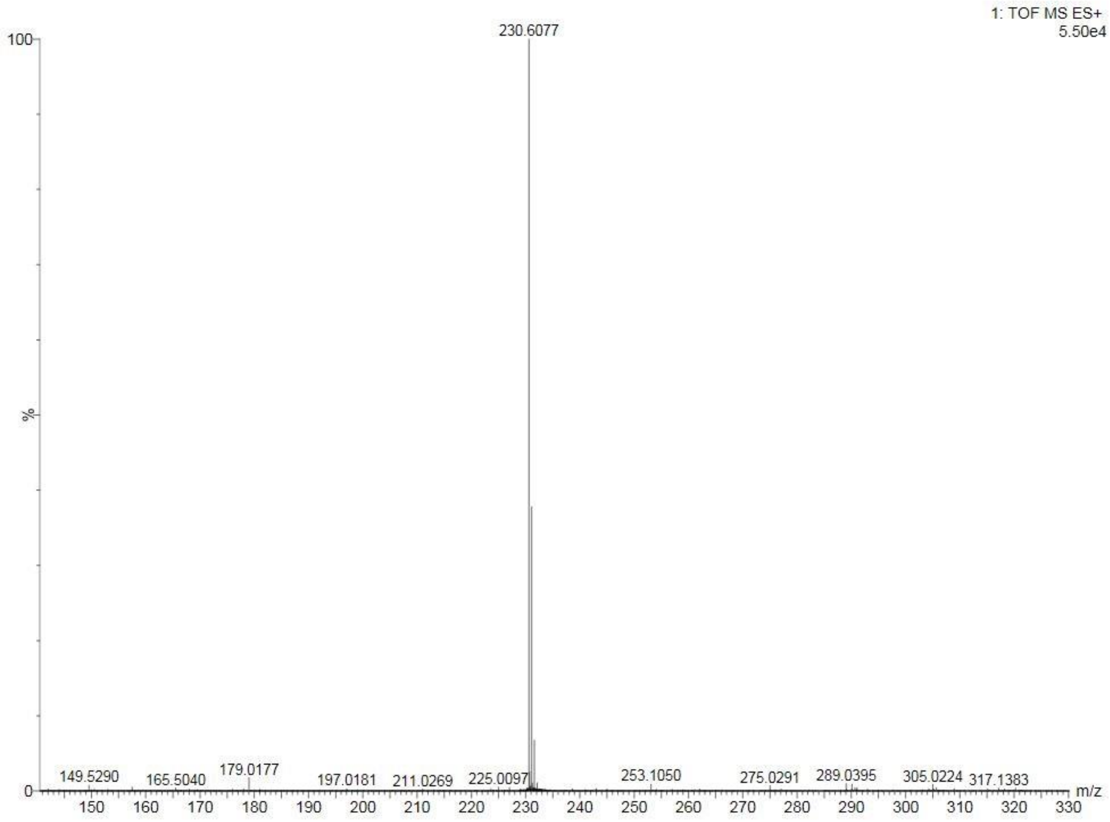

Fig. S18 HRMS spectrum of **compound 2**.

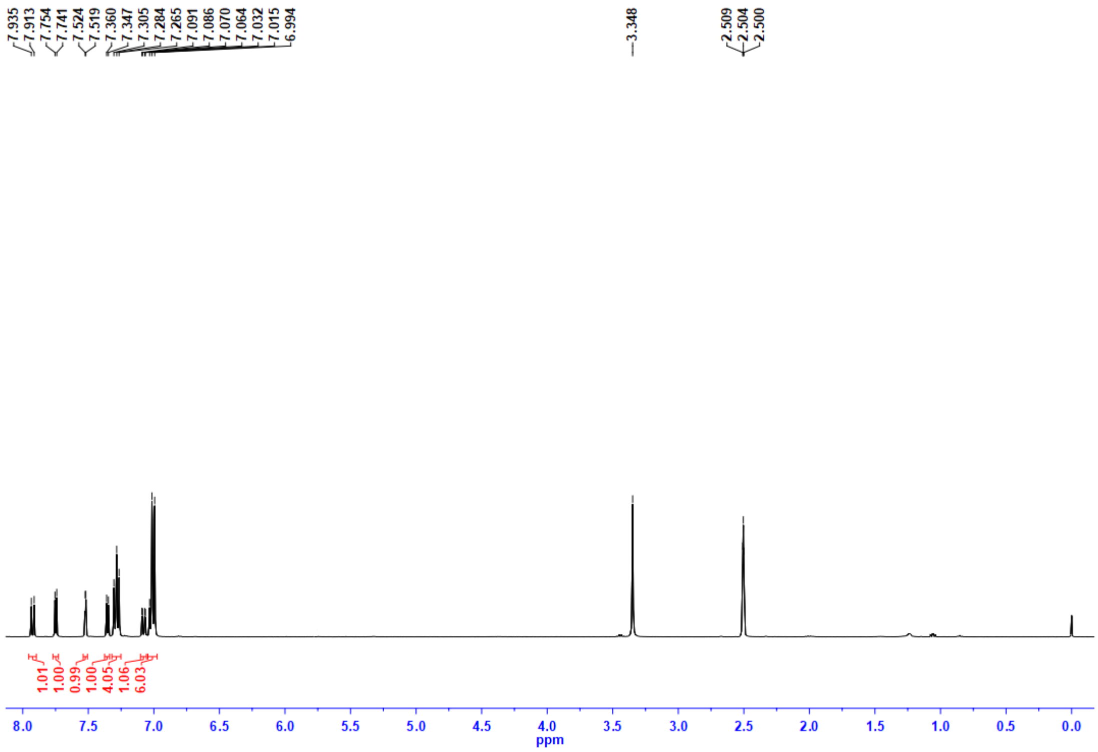

Fig. S19 ^{1}H NMR spectrum of **compound 4** in DMSO-d_6.

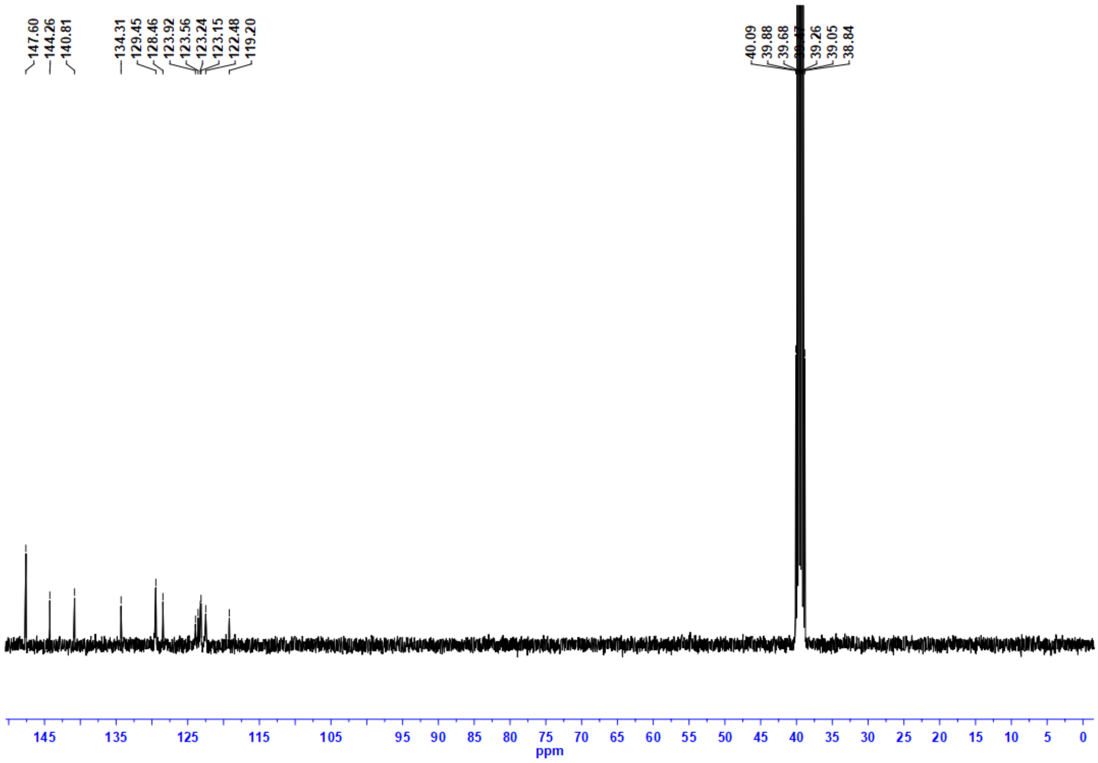

Fig. S20 ^{13}C NMR spectrum of **compound 4** in DMSO-d_6.

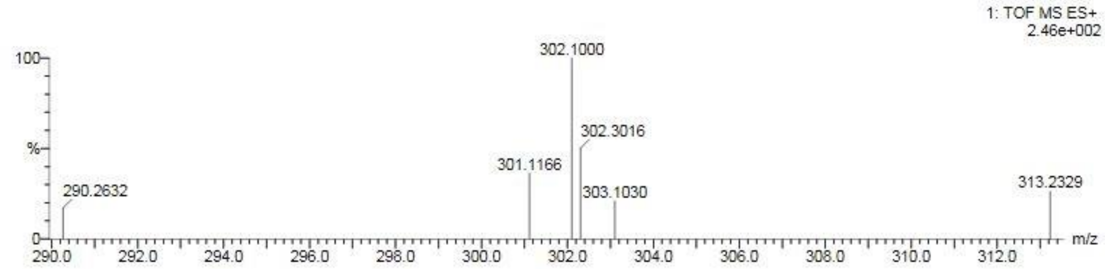

Minimum:				-1.5			
Maximum:		5.0	5.0	50.0			
Mass	Calc. Mass	mDa	PPM	DBE	i-FIT	i-FIT (Norm)	Formula
302.1000	302.1003	-0.3	-1.0	13.5	25.5	0.0	C20 H16 N S

Fig. S21 HRMS spectrum of **compound 4**.

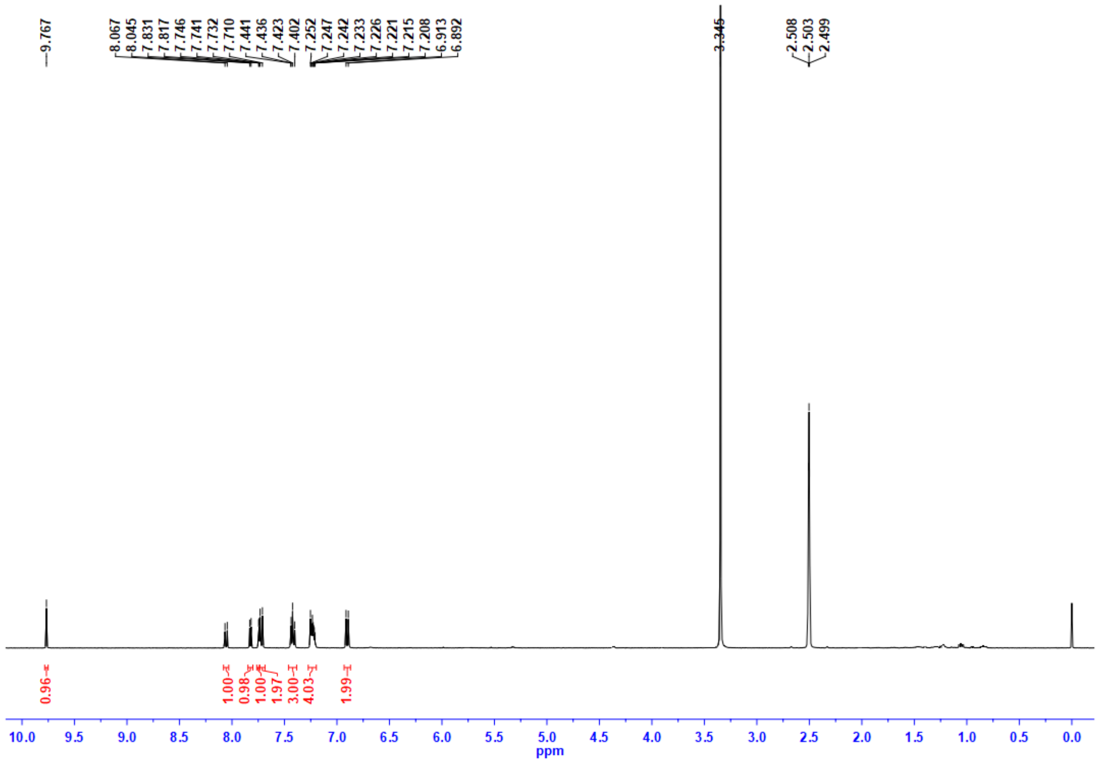

Fig. S22 ^{1}H NMR spectrum of **compound 5** in DMSO-d_6.

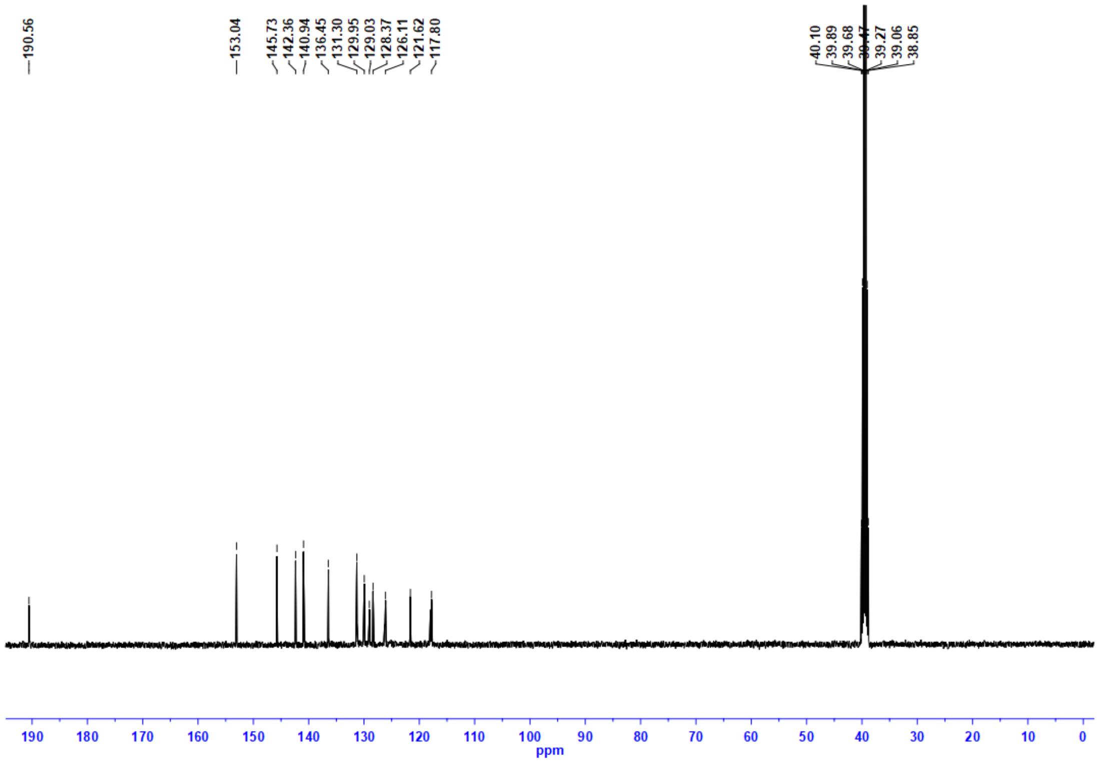

Fig. S23 ^{13}C NMR spectrum of **compound 5** in DMSO-d_6.

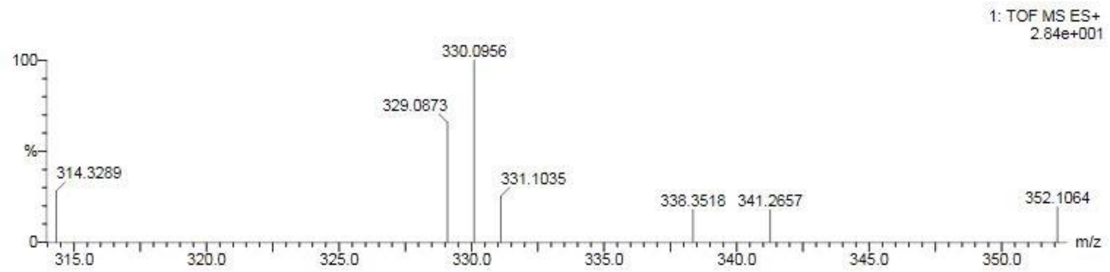

Minimum:				-1.5			
Maximum:		5.0	5.0	50.0			
Mass	Calc. Mass	mDa	PPM	DBE	i-FIT	i-FIT (Norm)	Formula
330.0956	330.0953	0.3	0.9	14.5	9.6	0.0	C21 H16 N O S

Fig. S24 HRMS spectrum of **compound 5**.

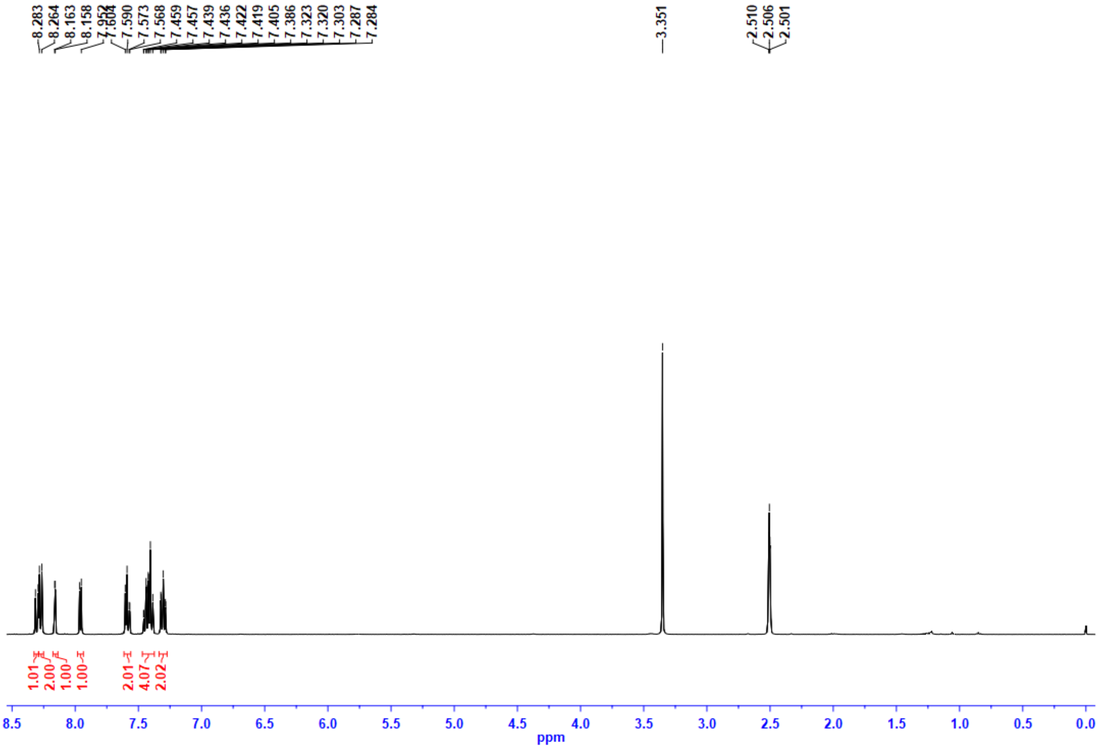

Fig. S25 ^{1}H NMR spectrum of **compound 6** in DMSO-d_6.

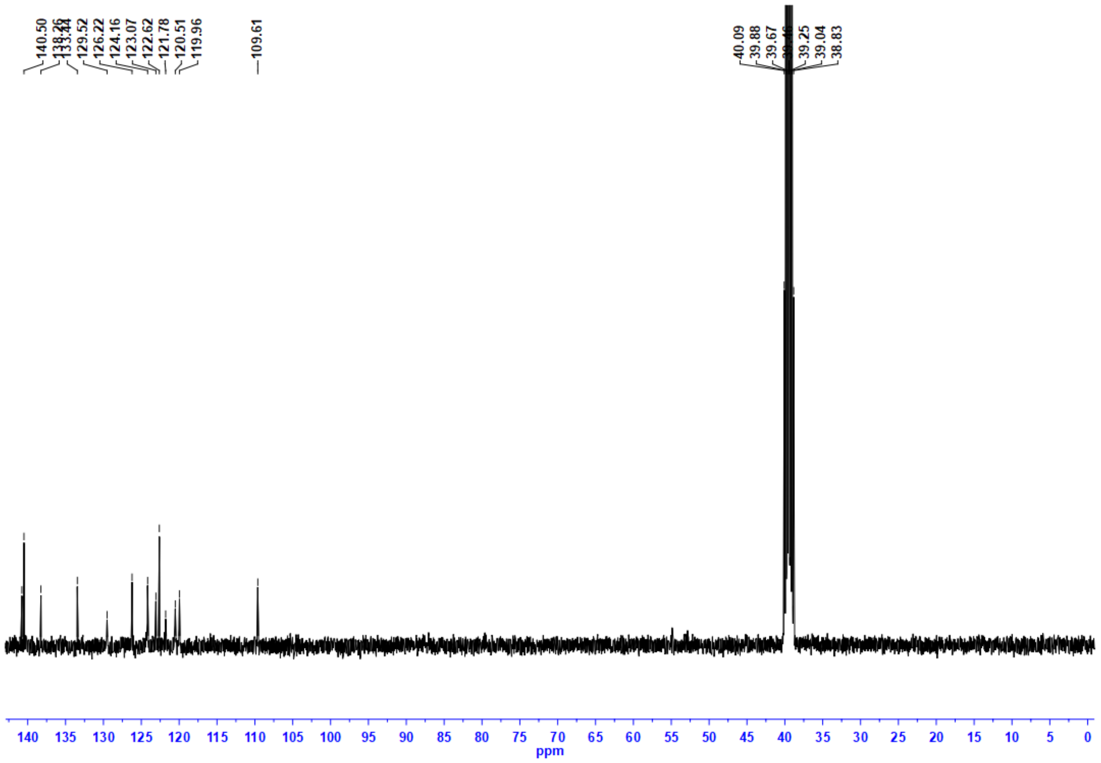

Fig. S26 ^{13}C NMR spectrum of **compound 6** in DMSO-d_6.

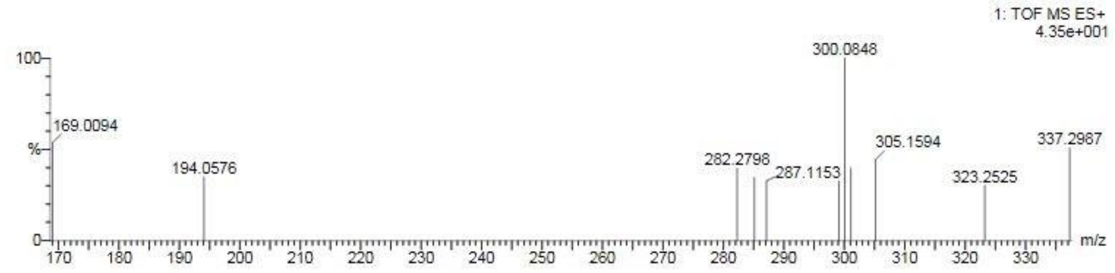

Minimum:				-1.5			
Maximum:		5.0	5.0	50.0			
Mass	Calc. Mass	mDa	PPM	DBE	i-FIT	i-FIT (Norm)	Formula
300.0848	300.0847	0.1	0.3	14.5	11.1	0.0	C20 H14 N S

Fig. S27 HRMS spectrum of **compound 6**.

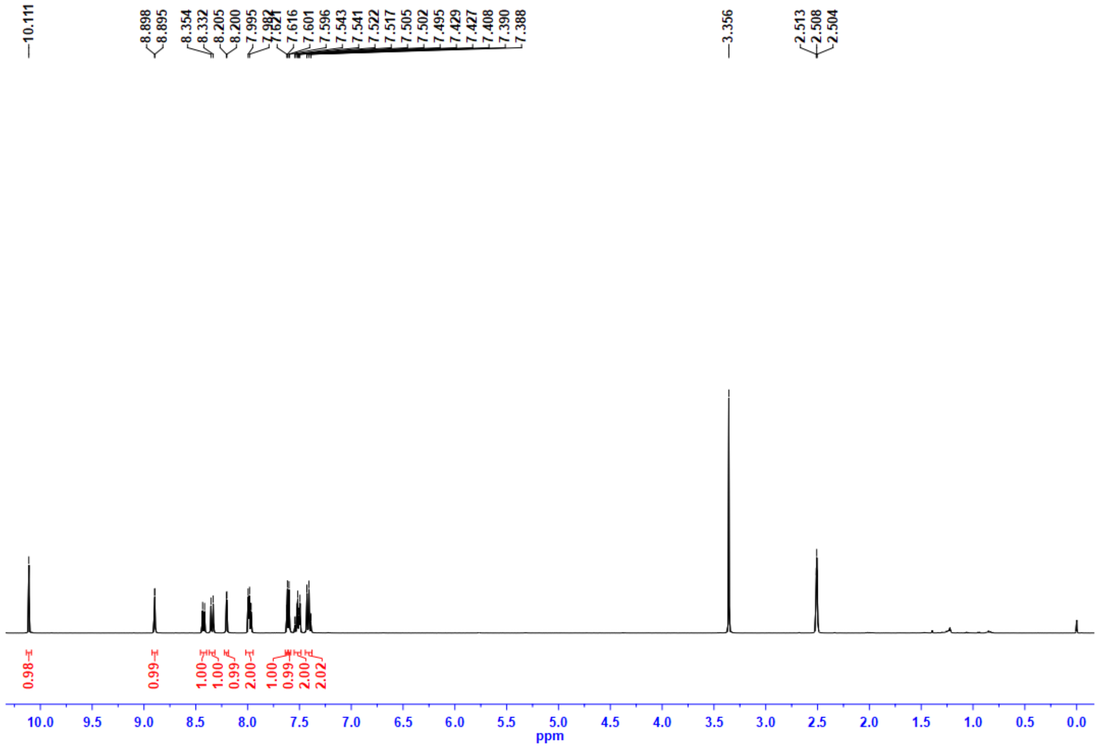

Fig. S28 ^{1}H NMR spectrum of **compound 7** in DMSO-d_6.

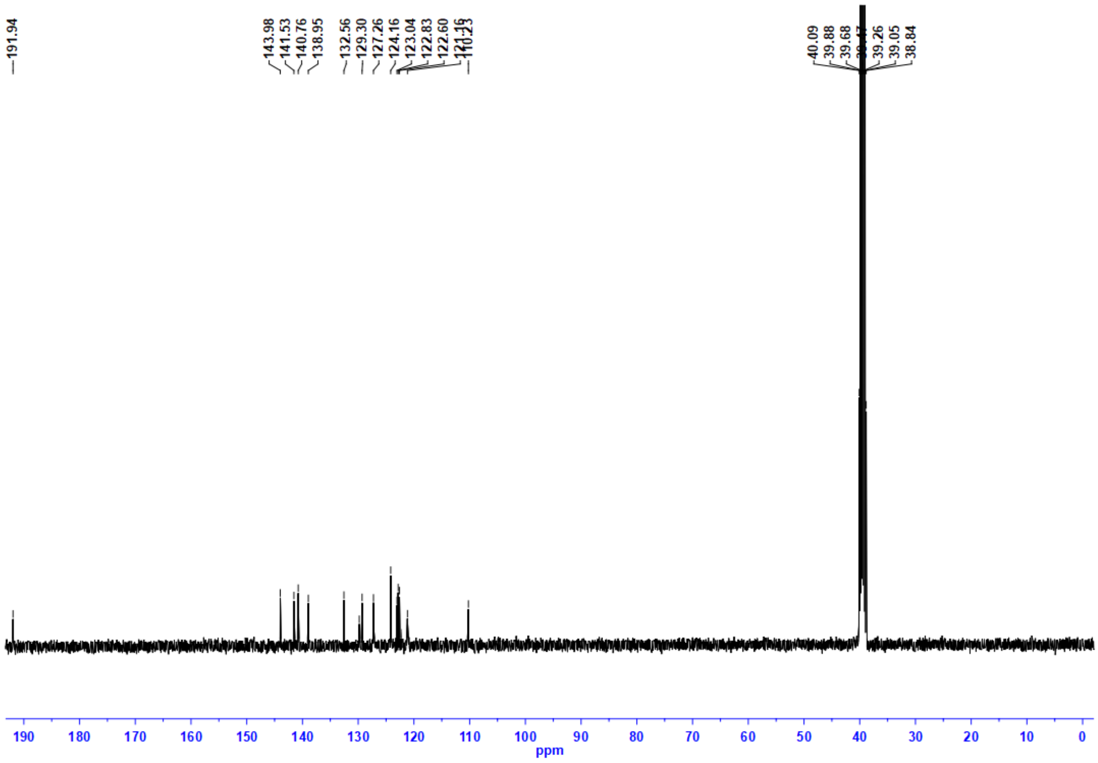

Fig. S29 ^{13}C NMR spectrum of **compound 7** in DMSO-d_6.

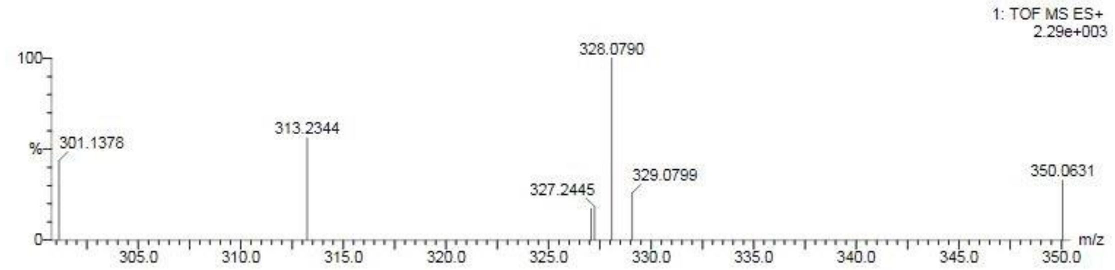

	Mass	Calc. Mass	mDa	PPM	DBE	i-FIT	i-FIT (Norm)	Formula
Minimum:					-1.5			
Maximum:			5.0	5.0	50.0			
	328.0790	328.0796	-0.6	-1.8	15.5	25.1	0.0	C21 H14 N O S

Fig. S30 HRMS spectrum of **compound 7**.

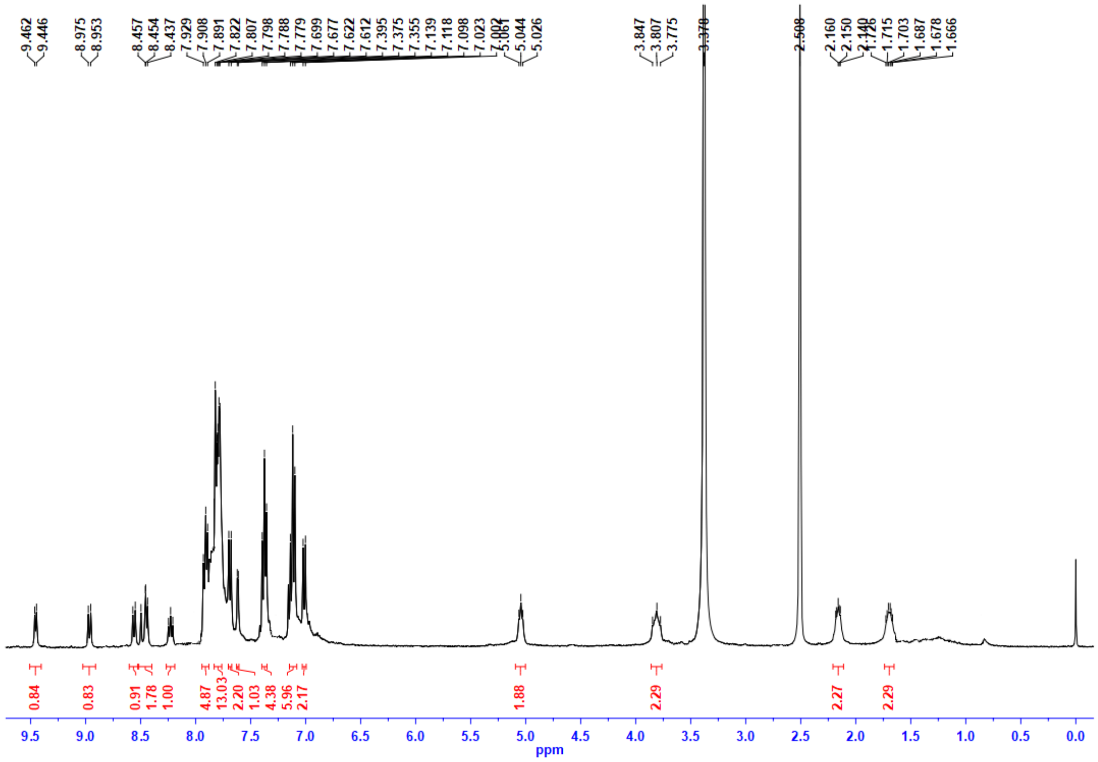

Fig. S31 ^{1}H NMR spectrum of **LOCK-0** in DMSO-d_6.

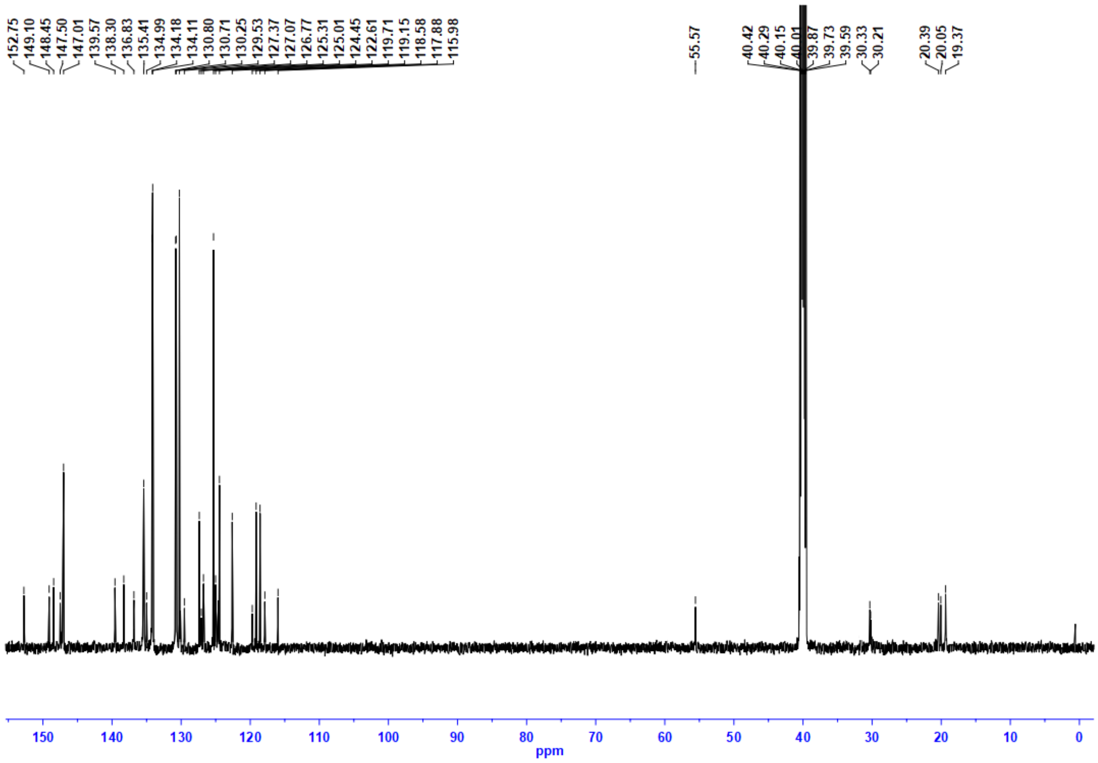

Fig. S32 ^{13}C NMR spectrum of **LOCK-0** in DMSO-d_6.

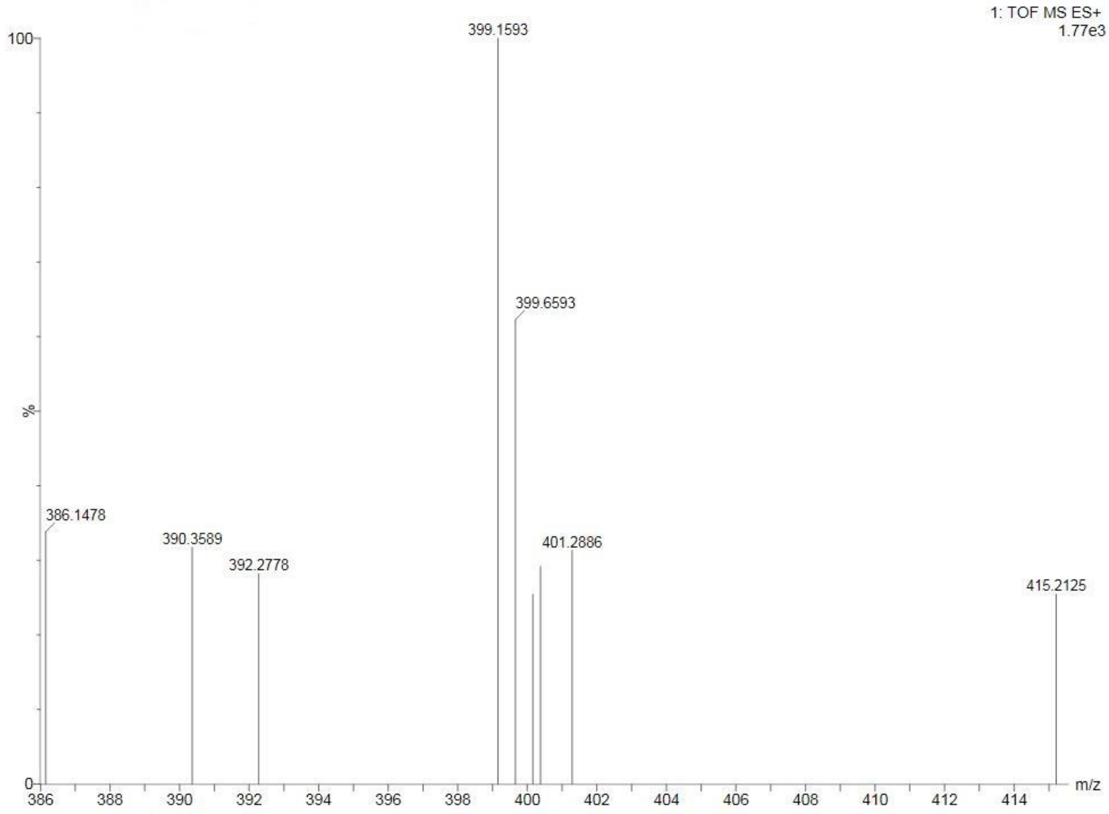

Fig. S33 HRMS spectrum of **LOCK-0**.

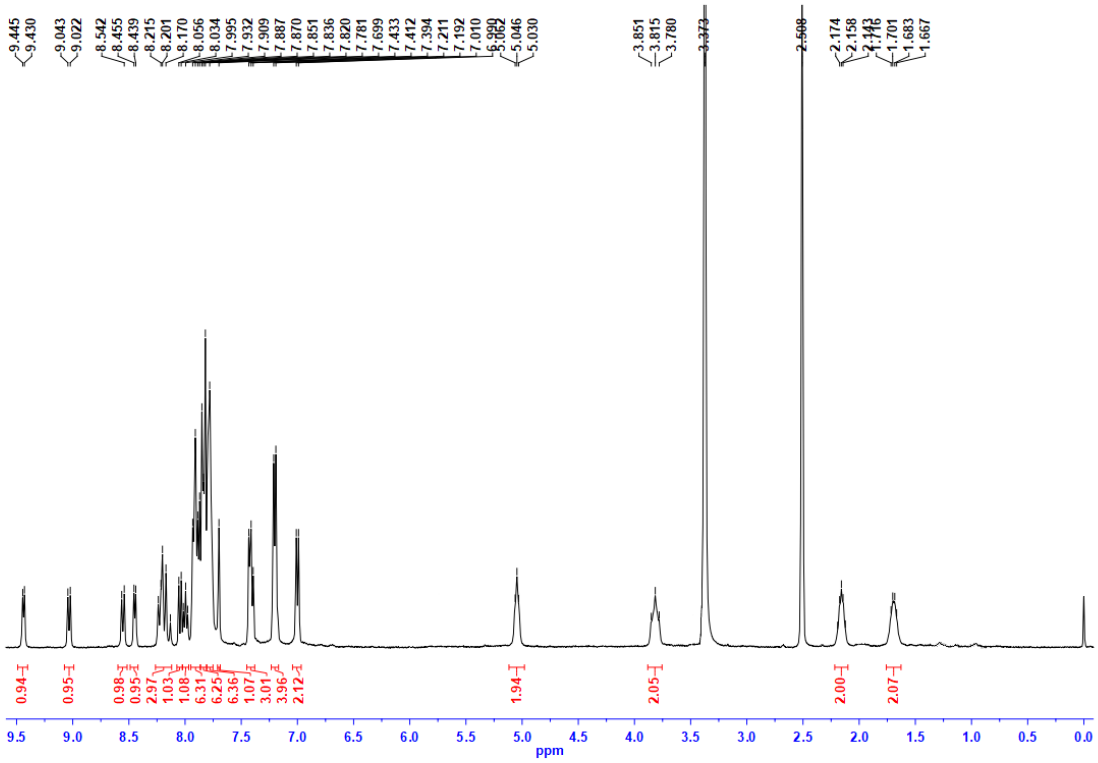

Fig. S34 ^{1}H NMR spectrum of **LOCK-1** in DMSO-d_6.

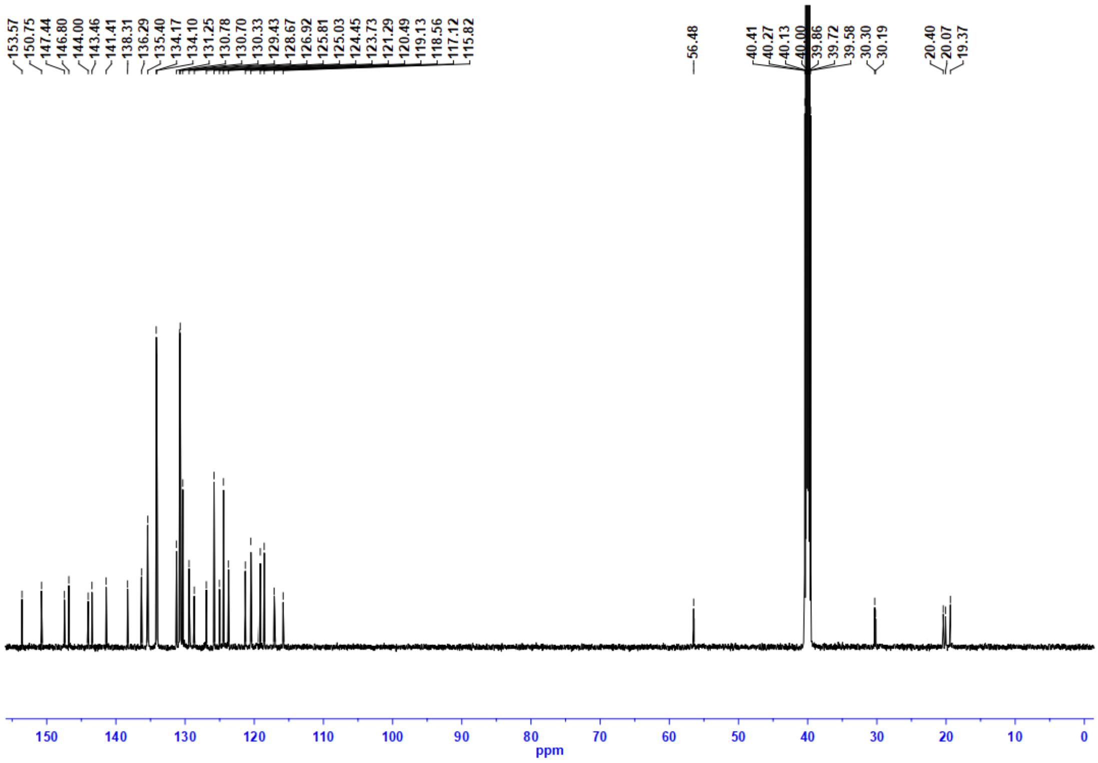

Fig. S35 ^{13}C NMR spectrum of **LOCK-1** in DMSO-d_6.

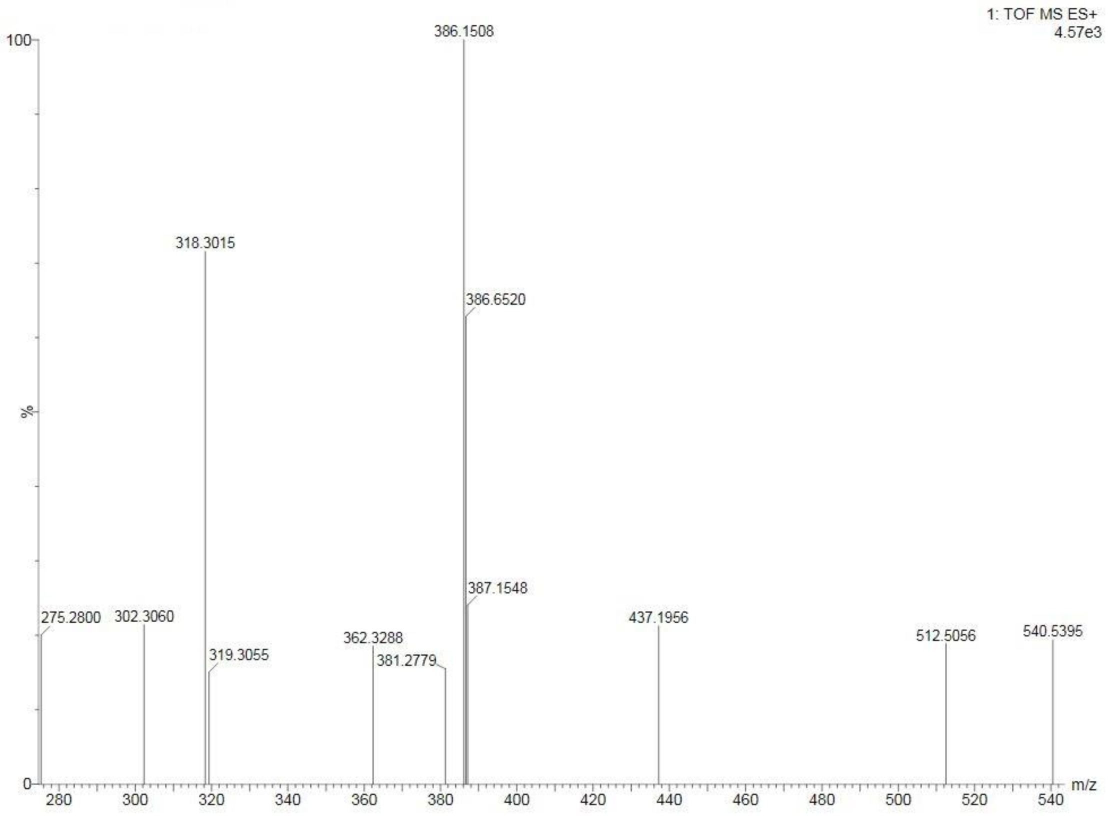

Fig. S36 HRMS spectrum of **LOCK-1**.

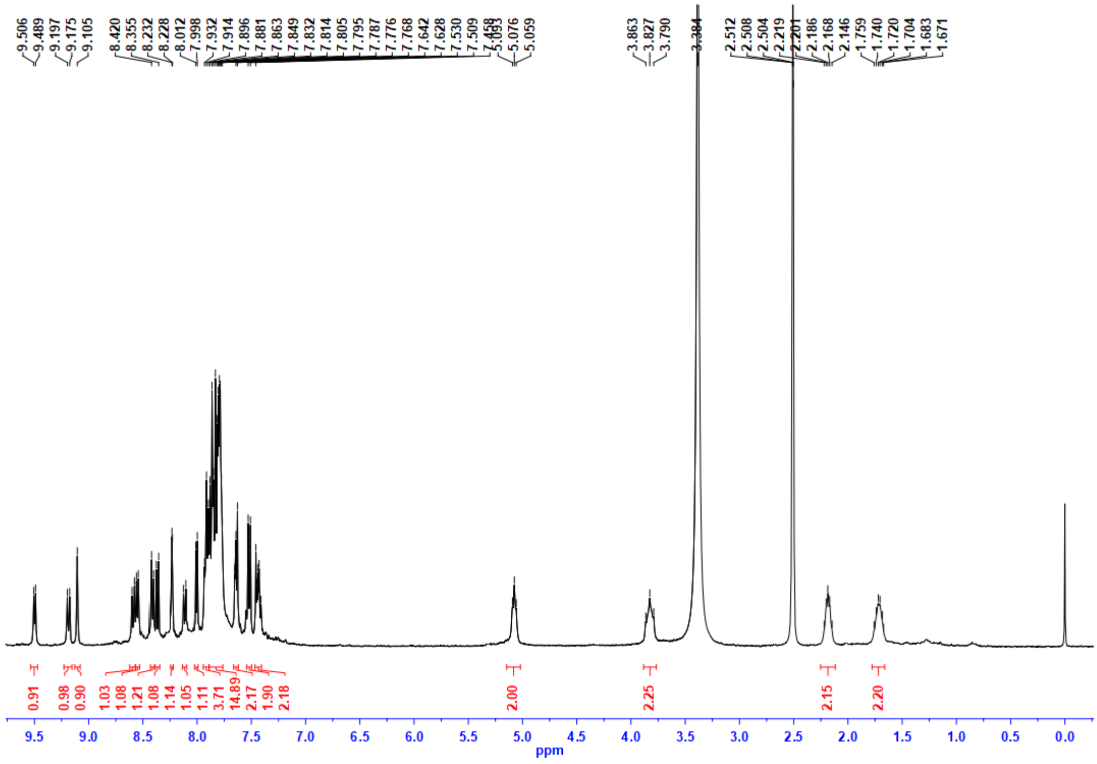

Fig. S37 ^{1}H NMR spectrum of **LOCK-2** in DMSO-d_6.

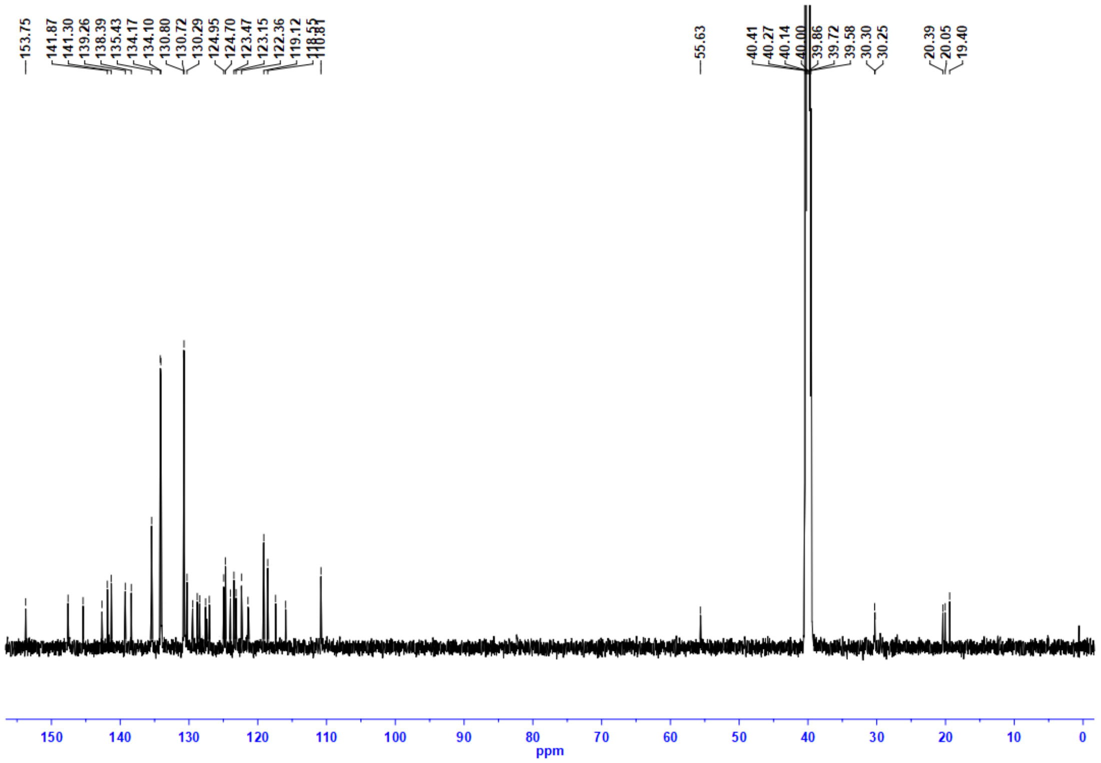

Fig. S38 ^{13}C NMR spectrum of **LOCK-2** in DMSO-d_6.

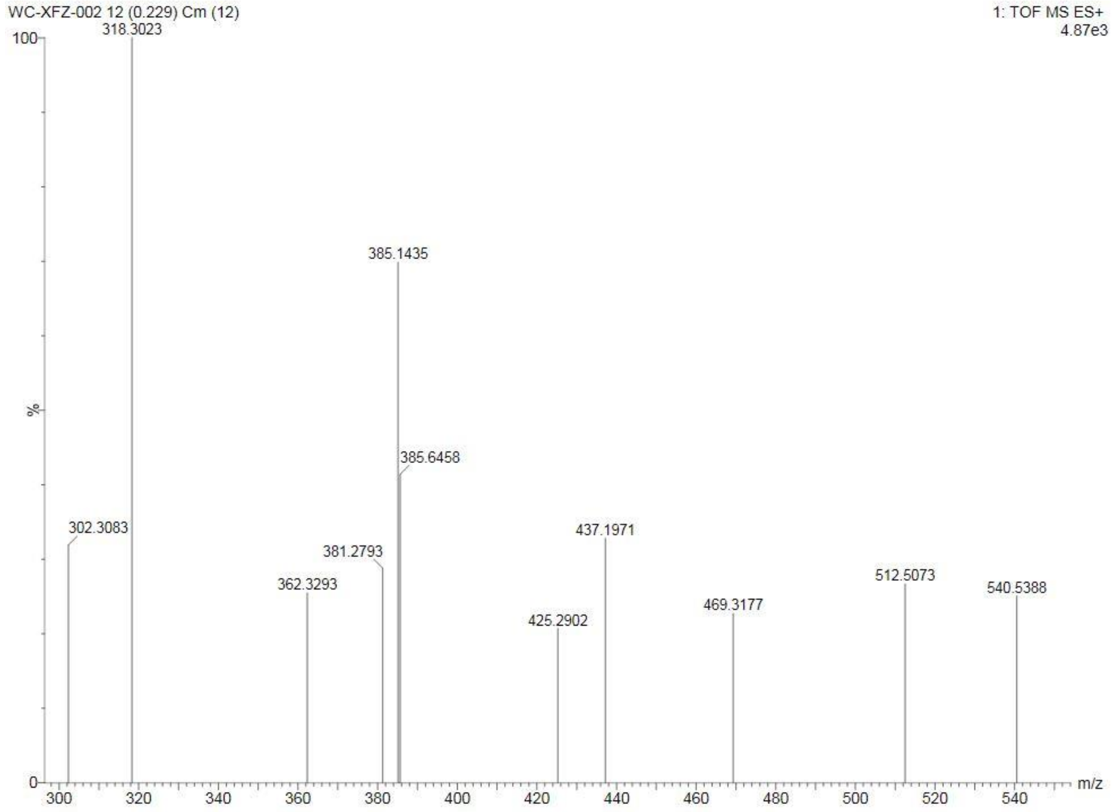

Fig. S39 HRMS spectrum of **LOCK-2**.

Table S1 Energy levels and energy gaps of HOMO and LUMO for **LOCK-0**, **LOCK-1** and **LOCK-2**.

	**LOCK-0**	**LOCK-1**	**LOCK-2**
LUMO (eV)	-2.26	-2.25	-2.29
HOMO (eV)	-6.24	-6.34	-6.71
ΔE_g (eV)	3.98	4.09	4.42

Table S2 Electronic excitation energy levels of singlet and triplet states for **LOCK-0**.

State	Energy level (eV)	State	Energy level (eV)	ΔE_{S1Tn} (eV)
S_1	2.2137	T_1	1.0687	1.1450
S_2	3.4545	T_2	2.3243	
S_3	3.9064	T_3	2.9496	
S_4	4.0416	T_4	3.0448	
S_5	4.3248	T_5	3.3669	
S_6	4.3825	T_6	3.4607	

Table S3 Electronic excitation energy levels of singlet and triplet states for **LOCK-1**.

State	Energy level (eV)	State	Energy level (eV)	ΔE_{S1Tn} (eV)
S_1	2.3236	T_1	1.1727	1.1509
S_2	3.5939	T_2	2.4849	
S_3	3.7627	T_3	3.0671	
S_4	4.1404	T_4	3.1305	
S_5	4.2083	T_5	3.3135	
S_6	4.4050	T_6	3.3817	

Table S4 Electronic excitation energy levels of singlet and triplet states for **LOCK-2**.

State	Energy level (eV)	State	Energy level (eV)	ΔE_{S1Tn} (eV)
S_1	2.7029	T_1	1.4350	1.2679
S_2	3.6480	T_2	2.5572	0.1457
S_3	3.7773	T_3	3.1027	
S_4	4.0670	T_4	3.1621	
S_5	4.1029	T_5	3.2589	
S_6	4.3748	T_6	3.5185	

References

1 H. Huang and Y. Tian, *Chem. Commun.*, 2018, **54**, 12198-12201.
2 H. E. Okda, S. El Sayed, R. C. M. Ferreira, R. C. R. Gonçalves, S. P. G. Costa, M. M. M. Raposo, R. Martínez-Máñez and F. Sancenón, *New J. Chem.*, 2019, **43**, 7393-7402.
